# SARS: Systematic Review of Treatment Effects

**DOI:** 10.1371/journal.pmed.0030343

**Published:** 2006-09-12

**Authors:** Lauren J Stockman, Richard Bellamy, Paul Garner

**Affiliations:** 1 Centers for Disease Control and Prevention, Respiratory and Enteric Viruses Branch, Atlanta, Georgia, United States of America; 2 Department of Veterans' Affairs, Atlanta Research and Education Foundation, Decatur, Georgia, United States of America; 3 James Cook University Hospital, Middlesbrough, United Kingdom; 4 Liverpool School of Tropical Medicine, Liverpool, United Kingdom; Mount Sinai Hospital, Canada

## Abstract

**Background:**

The SARS outbreak of 2002–2003 presented clinicians with a new, life-threatening disease for which they had no experience in treating and no research on the effectiveness of treatment options. The World Health Organization (WHO) expert panel on SARS treatment requested a systematic review and comprehensive summary of treatments used for SARS-infected patients in order to guide future treatment and identify priorities for research.

**Methods and Findings:**

In response to the WHO request we conducted a systematic review of the published literature on ribavirin, corticosteroids, lopinavir and ritonavir (LPV/r), type I interferon (IFN), intravenous immunoglobulin (IVIG), and SARS convalescent plasma from both in vitro studies and in SARS patients. We also searched for clinical trial evidence of treatment for acute respiratory distress syndrome. Sources of data were the literature databases MEDLINE, EMBASE, BIOSIS, and the Cochrane Central Register of Controlled Trials (CENTRAL) up to February 2005. Data from publications were extracted and evidence within studies was classified using predefined criteria. In total, 54 SARS treatment studies, 15 in vitro studies, and three acute respiratory distress syndrome studies met our inclusion criteria. Within in vitro studies, ribavirin, lopinavir, and type I IFN showed inhibition of SARS-CoV in tissue culture. In SARS-infected patient reports on ribavirin, 26 studies were classified as inconclusive, and four showed possible harm. Seven studies of convalescent plasma or IVIG, three of IFN type I, and two of LPV/r were inconclusive. In 29 studies of steroid use, 25 were inconclusive and four were classified as causing possible harm.

**Conclusions:**

Despite an extensive literature reporting on SARS treatments, it was not possible to determine whether treatments benefited patients during the SARS outbreak. Some may have been harmful. Clinical trials should be designed to validate a standard protocol for dosage and timing, and to accrue data in real time during future outbreaks to monitor specific adverse effects and help inform treatment.

## Abstract 2

A systematic review and comprehensive summary of reported treatments of SARS-infected patients, requested by the WHO expert panel on SARS, could not determine whether any treatments benefited patients.

## Editors' Summary

**Background.:**

Severe acute respiratory syndrome (SARS) is caused by a virus; the main symptoms are pneumonia and fever. The virus is usually passed on when people sneeze or cough. SARS became a much-talked about disease in 2003, when over 8,000 cases and 774 deaths occurred worldwide. The situation was alarming, because the first-ever cases had only just appeared in 2002, in China, so the best way to treat this new disease was unknown. Not many drugs are effective against viruses, and all doctors can usually do with a viral disease is to treat specific symptoms (e.g., fever and inflammation) and rely on the body's own immune system to fight off the virus itself. However, in recent years a number of antiviral drugs have been developed (for example, several are in use against HIV/AIDS), so there was hope that some of them might be active against SARS. Steroids were also often used in SARS treatment to try to reduce the inflammation of the lungs. In order to find out which, if any, of the potential treatments for SARS were effective, a number of research studies were carried out, both during and since the recent outbreak.

**Why Was This Study Done?:**

Health care decisions should be based on all the information that is available. It is important to try to bring together all the reliable evidence that exists on each possible treatment for a disease. The process of doing so is called a systematic review. In October 2003 the World Health Organization (WHO) established an International SARS Treatment Study Group, consisting of experts experienced in treating patients with SARS. The group recommended a systematic review of potential treatments for SARS. In particular, it was considered important to summarise the available evidence on the use of certain antiviral drugs (ribavirin, lopinavir, and ritonavir), steroids, and proteins called immunoglobulins, which are found naturally in human blood. The WHO group wanted to know how these treatments affected the virus outside the body (“in vitro”) and whether it helped the condition of patients and reduced the death rate, particularly in those patients who developed the dangerous complication called acute respiratory distress syndrome (ARDS). This study is a systematic review conducted in response to the WHO request.

**What Did the Researchers Do and Find?:**

They did no new work with patients or in the laboratory. Instead they conducted a comprehensive search of the scientific and medical literature for published studies that fitted their carefully predefined selection criteria. They found 54 SARS treatment studies, 15 in vitro studies, and three ARDS studies that met these criteria. Some of the in vitro studies with the antiviral drugs found that a particular drug reduced the reproduction rate of the viruses, but most of the studies of these drugs in patients were inconclusive. Of 29 studies on steroid use, 25 were inconclusive and four found that the treatment caused possible harm.

**What Do These Findings Mean?:**

From the published studies, it is not possible to say whether any of the treatments used against SARS were effective. No cases of SARS have been reported since 2004 but it is always possible that the same or a similar virus might cause outbreaks in the future. It is disappointing that none of the research on SARS is likely to be useful in helping to decide on the best treatments to use in such an outbreak. The authors discuss the weaknesses of the studies they found and urge that more effective methods of research be applied, in a timely fashion, in any similar outbreaks in the future. While the systematic review suggests that we do not know which if any of the potential treatments against SARS are effective, its recommendations mean that researchers should at least be better prepared to learn from potential future outbreaks.

**Additional Information.:**

Please access these Web sites via the online version of this summary at http://dx.doi.org/10.1371/journal.pmed.0030343.

- Wikipedia entry on SARS (Wikipedia is a free online encyclopedia that anyone can edit)
- MedlinePlus pages on SARS
- Wikipedia entry on systematic reviews, which includes links to other Web sites where more detailed information may be found

## Introduction

The severe acute respiratory syndrome (SARS) is a febrile respiratory illness primarily transmitted by respiratory droplets or close personal contact. A global outbreak of SARS between March 2003 and July 2003 caused over 8,000 probable or confirmed cases and 774 deaths [1]. The causative organism has been identified as a novel coronavirus (SARS-CoV) [2–4]. The overall mortality during the outbreak was estimated at 9.6% [5,6]. The overriding clinical feature of SARS is the rapidity with which many patients develop symptoms of acute respiratory distress syndrome (ARDS). This complication occurred in approximately 16% of all patients with SARS, and when it occurred was associated with a mortality rate of 50% [7,8].

At the time of the SARS epidemic it was not known what treatments would reduce SARS-related illness and deaths. Because the urgency of the international outbreak did not allow time for efficacy studies, physicians in Canada and Hong Kong treated the earliest patients with intravenous ribavirin, based on its broad-spectrum antiviral activity [9,10]. Corticosteroids and immune-modulating agents were often prescribed empirically. Soon after SARS-CoV was identified as the causative agent, antiviral screening programs were initiated; these programs reported several antiviral agents that inhibited SARS-CoV replication in vitro. These results led to the experimental use of protease inhibitors and interferon alpha (IFN-α) in the treatment of patients.

The most commonly used treatments for SARS are associated with adverse effects when used for other conditions (Table S1). In October 2003, the WHO established an International SARS Treatment Study Group, consisting of experts experienced in managing SARS. The group recommended a systematic review of potential treatment options to identify the targets for proper evaluation in trials should the disease recur [11]. This paper reports on this systematic review designed to summarise available evidence on the effects of ribavirin, lopinavir and ritonavir (LPV/r), corticosteroids, type I IFN, intravenous immunoglobulin (IVIG), or convalescent plasma in relation to (1) SARS-CoV replication inhibition in vitro; (2) mortality or morbidity in SARS patients; and (3) effects on ARDS in adult patients.

## Methods

We prepared a protocol that defined our scope, inclusion criteria, and outcomes to be assessed. The interventions we included were defined by the WHO: ribavirin, LPV/r, corticosteroids, type I IFN, convalescent plasma, or IVIG.

The types of study we included were: (1) in vitro studies, in which the authors examined inhibition of SARS-CoV viral replication, *and* data from an assay in human or animal cell line; (2) in vivo studies, which included randomised controlled trial (RCT), *or* prospective uncontrolled study design, *or* retrospective cohort design, *or* case-control design, *or* a case series, *and* patients treated for SARS, *and* ten or more patients; and (3) studies of ARDS that included RCT, *or* systematic review, *and* treatment for ARDS or acute lung injury, *and* 20 or more patients. In February 2005, we systematically searched the literature databases MEDLINE, EMBASE, BIOSIS, and the Cochrane Central Register of Controlled Trials (CENTRAL) for articles that included the selected treatments (Table S2).

The full text of each identified study was retrieved and each was independently reviewed by two authors (LS and RB). Publications in Chinese were selected after review of the English abstract. Unpublished data were not sought, as the task of summarising existing published data was extensive and the International SARS Treatment Group indicated that much of the clinical data had already been published. We used the QUOROM checklist to help ensure the quality of this review (Table S3).

Data from the full text of studies in English were extracted independently by two authors (LS and RB). Data from the Chinese literature were extracted with the assistance of a translator. Because the Chinese articles were reviewed by only one author, the consistency of the translated information with that from English articles was maintained by subsequent discussion with the translator to verify the extracted data.

We established explicit criteria to assess the level of evidence for each human treatment study (Box 1). Since the treatments chosen for evaluation were often given in combination, evidence was classified by the treatment that was given to all patients in the cohort or given to some with the author's intention of studying its effects. If putative effects within a study included several drugs, then we extracted data for each intervention. The level of evidence was independently classified by two authors (LS and RB). Chinese studies were appraised and classified in the same way using translated information extracted from each report. Discrepancies were resolved by consensus.

**Box 1. Categories of Evidence Defined for In Vivo Studies of Treatments in SARS Patients**

“*Inconclusive*” if a study could not be used to inform a decision about treatment efficacy due to having either outcomes which were not reported consistently, an inconsistent treatment regimen, no control group or a control group which was a likely source of bias. A control group was considered a likely source of bias if there were differences in co-morbidities, sex, age and markers of severe disease compared to the treatment group.

“Possible harm” if a study reported adverse effects of treatment that were consistent with adverse effects reported with the use of the drug in the treatment of other conditions. Evidence of direct causality was not required. A study could be classified as suggesting possible harm from the drug even if the study had methodological weaknesses.

“Possible benefit” if a study had evidence of benefit for an important outcome measure which was recorded consistently (e.g., case fatality, need for mechanical ventilation, duration of hospitalization, frequency of ARDS) in patients treated in a defined way compared to a valid control group. A control group was considered valid if randomized, or if patient characteristics and illness severity were comparable to the treatment group. Evidence of direct causality was not required.

“Definite harm” if a study contained statistically significant evidence of harm demonstrated in a double-blind randomized trial, which did not contain serious methodological weaknesses.

“Definite benefit” if a study contained statistically significant evidence of harm demonstrated in a double-blind randomized trial, which did not contain serious methodological weaknesses.

## Results

**Figure 1:**
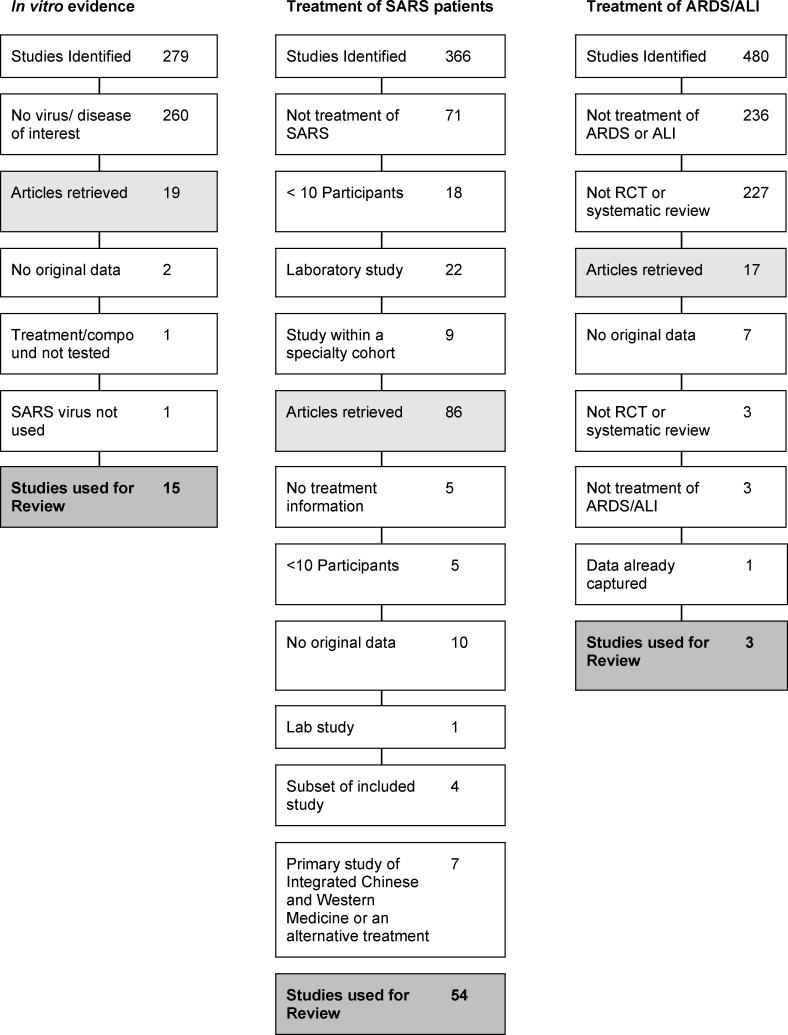
Process of Study Exclusion for Each Objective Category

In vitro evidence was available in 15 studies. Clinical evidence of SARS treatment in humans was reported in 54 studies (37 in English, 17 in Chinese). Three studies addressed treatment of ARDS (Figure 1).

### Ribavirin

#### In vitro.

We found six studies that described the antiviral effect of ribavirin in vitro (Table S4); four showed an antiviral effect (Table S5). A synergistic antiviral effect between ribavirin and type I IFN (IFN-β1a or leukocytic IFN-α) was described in two studies performed in human cell lines and Vero cell lines [12,13].

#### In SARS patients.

We found 24 studies that described ribavirin treatment in cohorts larger than ten patients (Table S6). Our formal assessment classified 20 studies as “inconclusive,” due to study design or because the effect of ribavirin could not be distinguished from the effects of other treatments (such as steroids and antiviral drugs). Four publications presented evidence of possible harm (14–17). Three of these studies, each of which included over 100 patients, documented a fall in haemoglobin levels after ribavirin treatment when compared to levels in patients before treatment [14–16]. Of patients treated with ribavirin, 49/138 to 67/110 (36%–61%) developed haemolytic anaemia, a recognised complication with this drug, although it is not possible to rule out the possibility that SARS-CoV infection caused the haemolytic anaemia, as there is no control group. One study noted that over 29% of SARS patients had some degree of liver dysfunction indicated by ALT levels higher than normal, and the number of patients with this complication increased to over 75% after ribavirin treatment (Table S7) [17].

In the Chinese literature six additional reports described patients with SARS treated with ribavirin (often with steroids). These six reports were determined to be inconclusive in the evaluation of treatment for SARS (Tables S8 and S9).

### LPV/r

#### In vitro.

Of three studies, two demonstrated that lopinavir inhibits cytopathic effects of SARS-CoV in fetal rhesus monkey kidney cells (Table S4). One study showed detectable but reduced activity in Vero-E6 cells [13], and one study concluded that neither lopinavir nor ritonavir had an effect [18]. A synergistic effect of lopinavir with ribavirin has been reported (Table S5).

#### In SARS patients.

We found two studies of LPV/r (lopinavir 400 mg with ritonavir 100 mg orally every 12 h) in cohorts larger than ten patients (Table S6). Patients also received ribavirin and corticosteroids. LPV/r use was compared among three groups of patients: those who received it as an early SARS treatment, those who received it as a late treatment, and those who did not receive it at all.

When LPV/r was added as an initial treatment to ribavirin and corticosteroid therapy, the death rate was lower than among those who received ribavirin and corticosteroids (1/44 [2.3%] versus 99/634 [15.6%]; *p* < 0.05) [19]. A second study of this regimen reported fewer episodes of ARDS or death compared with historical controls who had not received LPV/r (1/41 [2.4%] versus 32/111 [28.8%]; *p* < 0.001) (Table S7) [20]. Both studies were determined to be inconclusive due to possible bias in the selection of control group or treatment allocation.

No additional studies were identified from the Chinese literature.

### Corticosteroids 

#### In vitro.

No studies were found on the cytopathic effect of corticosteroids alone against SARS-CoV. Corticosteroids act as immunomodulatory agents, and therefore studies to measure direct antiviral effects in vitro were not expected.

#### In SARS patients.

Fifteen articles examined corticosteroid treatment in ten or more patients. Of these cohorts 13 were also treated with ribavirin (Table S6). We determined that 13 of the 15 studies were inconclusive. Of these, in an uncontrolled and nonrandomised study, 95/107 (89%) of patients treated with high-dose methylprednisolone (0.5–1 mg/kg prednisolone on day 3 of illness, followed by hydrocortisolone 100 mg every 8 h, and pulse-doses of methylprednisolone 0.5 g IV for 3 d) after the first week of illness recovered from progressive lung disease (Table S7) [16].

Two studies contained evidence of possible harm from corticosteroids [21,22]. One measured SARS-CoV plasma viral load across time after fever onset in a randomized, double-blind, placebo-controlled trial; corticosteroid use within the first week of illness was associated with delayed viral clearance. The other study, which was case-controlled, found that patients with psychosis received higher cumulative doses of steroids than patients without psychosis (10,975 mg versus 6,780 mg; *p* = 0.017) [22].

In the Chinese literature, we found 14 reports in which steroids were used (Table S8 and Table S9). Twelve studies were inconclusive and two showed possible harm. One study reported diabetes onset associated with methylprednisolone treatment [23]. Another study (an uncontrolled, retrospective study of 40 SARS patients) reported avascular necrosis and osteoporosis among corticosteroid-treated SARS patients [24].

#### In ARDS patients.

Three clinical trials examined the effect of corticosteroids on mortality in patients with established ARDS (Table S10). In two trials, high-dose methylprednisolone given for approximately 2 d was not effective for early ARDS [25,26]. One small RCT that used a regimen of lower dose methylprednisolone (2 mg/kg per day), tapered after 2 wk, showed possible evidence of ARDS improvement (Table S11) [27].

### IFN Type I

#### In vitro.

Twelve in vitro studies with data on the antiviral effect of IFN type I have been reported, and all demonstrated an antiviral effect against SARS-CoV (six for IFN-α and ten for IFN-β) (Tables S4 and S5). Antiviral effects have been demonstrated in monkey (Vero; Vero-E6), fetal rhesus monkey kidney (fRhK-4), and human (Caco2, CL14, and HPEK) cell lines.

Three reports presented evidence that IFN-β was superior against SARS-CoV compared to IFN-α and found rIFN-α2 virtually ineffective against SARS-CoV compared to other IFNs [28]. Synergistic effects were reported for leukocytic IFN-α with ribavirin [13], IFN-β with ribavirin [12,13] and IFN-β with IFN-γ [28,29].

#### In SARS patients.

Two studies of IFN-α given with steroids and/or ribavirin were reported (Table S6). No significant difference was seen in outcome between IFN-α treatment group and those treated with other regimens. Results of both studies were inconclusive due to a lack of a consistent treatment regimen or suitable control group (Table S7).

In the Chinese literature, one additional study reported the use of IFN-α as part of a regimen that included ribavirin and steroids [30]. We determined this study to be inconclusive because a variety of treatments given masked the effect of IFN-α alone (Table S8 and Table S9).

### Convalescent Plasma or Immunoglobulin

#### In vitro.

No studies were found on the cytopathic effect of this treatment on SARS-CoV. Convalescent plasma and IVIG act as immunomodulatory agents and therefore studies to measure direct antiviral effects in vitro were not expected.

#### In SARS patients.

Five studies of either IVIG or convalescent plasma treatment given in addition to steroids and ribavirin were reported for treatment of SARS (Table S6). These studies were inconclusive, because the effect of convalescent plasma or IVIG could not be discerned from effects of patient comorbidities, stage of illness, or effect of other treatments (Table S7).

In the Chinese literature, two additional studies reported evidence on the effect of convalescent plasma as a treatment for SARS [30,31]. These studies were inconclusive (Table S8 and Table S9).

Evidence collected on the benefit or harm of drugs used to treat SARS is summarized in Table 1.

**Table 1:**
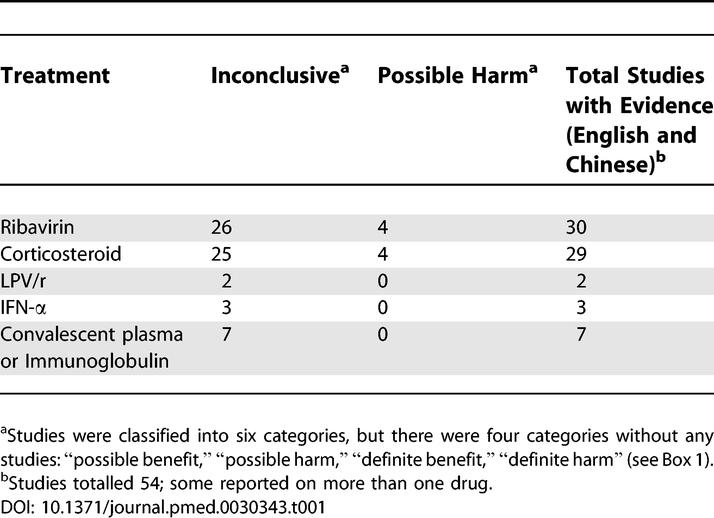
Summary of the Evidence for Benefit or Harm of Drugs Used to Treat SARS

## Discussion

The rapid spread and subsequent control of SARS precluded controlled clinical treatment trials during the outbreak of 2002–2003. In this report we summarize the results of a systematic evaluation of the findings from published reports of treatments used for SARS during the epidemic. Publications from the Chinese literature were included to capture as much evidence as possible. We developed specific criteria (Box 1) to look for large, obvious effects of benefit, adverse or poor outcomes, or evidence of potential benefit that could be used to prioritise future research of SARS treatments. A summary of this evidence in SARS patients is shown in Table 1.

Despite thirty reports of SARS-infected patients treated with ribavirin, there is no convincing evidence that it led to recovery. Haemolytic anaemia, a recognized side effect of this treatment, was observed in three studies. We would infer from these findings that any future use of ribavirin for SARS should be within the context of a controlled trial with close attention given to adverse effects.

Corticosteroids were commonly prescribed to SARS patients with worsening pulmonary disease or progressing abnormalities on chest X-rays. Treatment regimens varied widely but can be classified into two groups, early treatment and rescue treatment given at a later stage of illness. It is difficult to make a clear recommendation about whether corticosteroids should be used to treat SARS-associated lung injury in any stage of illness, particularly as the drug is immunosuppressive and may delay viral clearance if given before viral replication is controlled [21]. Of added concern are infectious complications, avascular necrosis, and steroid-induced psychosis—recognized adverse effects of corticosteroid use. Fungal superinfection and aspergillosis have been noted in case reports and autopsy findings of SARS patients given corticosteroids at high doses or for prolonged periods [32,33]. This review has found evidence of avascular necrosis and steroid-induced psychosis in SARS patients.

Seven studies of treatment with convalescent plasma or IVIG, three with IFN type I, and two with LPV/r were inconclusive by the criteria used in our analyses. Authors of four of the IVIG studies commented that patients seemed to improve upon treatment, but that more controlled trials of this approach are needed to provide evidence of an effect for SARS.

Important caveats should be considered in this review. Most of the studies of SARS patients were descriptions of the natural course of the disease and had not been designed to reliably assess the effects of the treatments used. Patient characteristics such as age and presence of diabetes mellitus have been associated with severe disease and can confound treatment effects. A diagnostic test for early SARS illness was not validated or widely available, and in general, treatment was initiated once patients fulfilled a clinical and epidemiological case definition. It is possible that the inclusion of patients without laboratory confirmation of SARS-CoV infection in this review could cause an underestimate of any true effect of antiviral treatment on SARS.

The variation in treatment regimens—particularly the wide range in doses, duration of therapy, and route of administration of ribavirin and corticosteroids—is a major obstacle to a clear interpretation of the data in this review. The nonstandardised collection of clinical information limits the conclusions that can be drawn from a retrospective analysis. We suggest that, in the event of a future outbreak of SARS-CoV or another novel agent, attempts be made to develop treatment protocols and to collect and contribute information for a standardized minimum dataset that could facilitate analysis of treatment outcomes among different settings. As observational studies pose problems of interpretation, the need is great for good-quality randomised trials, despite the difficulties in organising such trials.

## Supporting Information

Table S1 Rationale for Treatments and Recognized Adverse Effects (55 KB DOC)

Table S2 Method of Systematic Review (A) Search strategy, step 1: Select the treatments.
(B) Search strategy, step 2: Narrow the scope.
(C) Inclusion criteria and information sought from each study.
(45 KB DOC)

Table S3 QUOROM Statement (50 KB DOC)

Table S4 Description of SARS-CoV Replication Studies: Assay Type and Outcomes Measured (79 KB DOC)

Table S5 Results from SARS-CoV Replication Studies: Inhibition of SARS-CoV Replication (84 KB DOC)

Table S6 Description of Studies within SARS Patients (186 KB DOC)

Table S7 Results of Treatment within SARS Patients (English literature) (183 KB DOC)

Table S8 Description of Studies of SARS Patients (Chinese Literature) (108 KB DOC)

Table S9 Results of Treatment within SARS Patients (Chinese Literature) (93 KB DOC)

Table S10 Description of Studies of ARDS or ALI (50 KB DOC)

Table S11 Results of Treatment of ARDS or ALI (48 KB DOC)

## Abbreviations

ARDS: acute respiratory distress syndrome
IFN: interferon
IVIG: intravenous immunoglobulin
LPV/r: lopinavir and ritonavir
RCT: randomised controlled trial
SARS: severe acute respiratory syndrome
SARS-CoV: severe acute respiratory syndrome-associated coronavirus
