# Supplementary material for: SARS: Systematic Review of Treatment Effects
Source: PLoS Med. 2006 Sep 12;3(9):e343. doi: 10.1371/journal.pmed.0030343 (PMC1564166; doi:10.1371/journal.pmed.0030343)
Supplement: Supplementary file 1 [file pmed.0030343.st001.doc]

**Table S1. Rational for treatments and recognized adverse effects**

| **Treatment** | **Recognized adverse effects of treatment in other illnesses** |
| --- | --- |
| **Ribavirin** |  |
| Ribavirin, a nucleoside analogue which inhibits replication of a broad spectrum of viruses *in vitro.* | Haemolytic anemia and myocardial infarctions in patients with anemia *(1).* |
| **Corticosteroids** |  |
| Corticosteroids suppress the immune response. | Increased rate of infectious complications, psychic derangements, acute myopathy and osteonecrosis with high-doses *(2).* |
| **Lopinavir and Ritonavir** |  |
| Lopinavir and ritonavir are protease inhibitors which are used in the treatment of HIV infection. Ritonavir inhibits the metabolism of lopinavir, and thus helps maintain blood levels of lopinavir. | Side effects of combined treatment include diarrhea of mild to moderate severity. In HIV patients, the treatment has been associated with new-onset diabetes and hyperglycaemia *(3)* |
| **Type I Interferon** |  |
| Interferon alpha and beta are type I interferons that inhibit replication of RNA and DNA viruses. A pegylated (polyethylene glycol-conjugated) derivative of interferon alpha has a longer half life. | Flu-like symptoms are the most commonly reported side effect after long term treatment with interferon, however hypertension, myocardial infarctions and depression have been reported *(4)* |
| **Convalescent plasma or Immunoglobulin** |  |
| Convalescent plasma provides passive immunity for diseases such as Ebola. Specific immunoglobulin prepared from donors with high titres of the required antibody has been used to protect contacts exposed to hepatitis B virus, rabies, tetanus and varicella-zoster. | Commercial immune globulin intravenous human products have been reported to be associated with renal dysfunction, acute renal failure, osmotic nephrosis and death *(5)*. |

**References**

1	. Schering Corporation (2003) Rebetol (ribavirin, USP) Capsules. 2001. Available at: http://www.fda.gov/medwatch/SAFETY/2003/03May_PI/Rebetol_PI.pdf. Accessed 31 Oct 2005.

2. Pharmacia & Upjohn Company (2001) Solu-Medrol. Methylprednisolone sodium succinate for injection, USP. 2001. Available at: http://www.pfizer.com/pfizer/download/uspi_solumedrol.pdf. Accessed 31 Oct 2005.

3. Abbott Laboratories (2005) Kaletra (lopinavir/ritonavir) capsules (lopinavir/ritonavir) oral solution. Available at: http://www.fda.gov/cder/foi/label/2005/021226s016lbl.pdf. Accessed 31 Oct 2005.

4. Amgen (1997) Infergen (Interferon alfacon-1). Available at: http://www.fda.gov/cder/foi/label/1997/ifnamg100697lb.pdf. Accessed 31 Oct 2005.

5. Novartis Pharmaceuticals (2000) Immune globulin intravenous (human) Sandoglobulin. Available at: http://www.pharma.us.novartis.com/product/pi/pdf/sandoglobulin.pdf. Accessed: 31 Oct 2005.
