# Supplementary material for: SARS: Systematic Review of Treatment Effects
Source: PLoS Med. 2006 Sep 12;3(9):e343. doi: 10.1371/journal.pmed.0030343 (PMC1564166; doi:10.1371/journal.pmed.0030343)
Supplement: Supplementary file 2 [file pmed.0030343.st002.doc]

**Table S2(a). Search strategy (step 1: select the treatments)**

**Treatment**

Ribavirin *OR*

Lopinavir *or* Kaletra *or* ritonavir *OR*

Steroid *or* corticosteroid *or* prednisolone *or* prednisone *or* dexamethasone *or* dexametasone *or* hydrocortisone *OR*

Gamma-globulin *or* immune-globulin *or* immunoglobulin *or* γ-globulin *or* Sandoglobulin *or* convalescent plasma *or* FFP *or* serum *or* blood-derived *or* blood product *OR*

Interferon

Table S2(b). Search strategy (step 2: narrow the scope)

| ***In vitro* (Objective 1)** | **Treatment of SARS patients (Objective 2)** | **Treatment of ARDS (Objective 3)** |
| --- | --- | --- |
| Search 1 *AND*  SARS *or* severe acute respiratory syndrome *AND*  Viral replication *or* virus replication *or* *in vitro* | Search 1 *AND*  SARS *or* coronavirus *or* severe acute respiratory syndrome *AND*  Human | Search 1 *AND*  ARDS *or* acute respiratory distress syndrome *or* acute lung injury *or* systemic inflammatory response syndrome *or* SIRS *or* shock lung *AND*  Human *AND*  Randomized controlled trial *or* controlled clinical trial *or* randomized controlled trials *or* random allocation *or* double-blind method *or* single-blind method |

**Table S2(c). Inclusion criteria and information sought from each study.**

|  | ***in vitro*** | **Treatment of SARS patients** | **Treatment of ARDS** |
| --- | --- | --- | --- |
| **Inclusion criteria** | Inhibition of viral replication, AND  at least one compound of interest, AND  data from an assay in human or animal cell line. | RCT, *or* prospective uncontrolled study design, *or* retrospective cohort design, *or* case-control design *or*  a case series, AND  patients treated for SARS; AND  ten or more patients. | RCT, *or* systematic review,  AND  treatment for ARDS, *or* ALI, AND  twenty or more patients. |
| **Data extraction** | Language of publication, assay type, cell line, intervention tested, comparisons made, inferences made, overall conclusion. | Country of study, language of publication, study design, number of participants, study methods, age of participants, case definition applied for SARS diagnosis, stage of SARS disease, interventions used, number or amount treated with each intervention, outcomes reported, comparisons made, adverse effects measured or reported, inferences made by authors, basis for these inferences, critical appraisal of the results, overall conclusion. | Country of study, language of publication, study design, number of participants, study methods, stage of ALI/ARDS disease, interventions used, number or amount treated with each intervention, outcomes reported, comparisons made, adverse effects measured or reported, inferences made by authors, basis for these inferences, critical appraisal of the results, overall conclusion. |

ALI= Acute lung injury ARDS= Acute respiratory distress syndrome RCT= Randomized controlled trial
