# Supplementary material for: SARS: Systematic Review of Treatment Effects
Source: PLoS Med. 2006 Sep 12;3(9):e343. doi: 10.1371/journal.pmed.0030343 (PMC1564166; doi:10.1371/journal.pmed.0030343)
Supplement: Supplementary file 4 [file pmed.0030343.st004.doc]

## Table S4. Description of SARS-CoV replication studies: assay type and outcomes measured

| **Compound** | **Assay type** | **Combination** | **Outcomes Measured** | **Reference ID** |
| --- | --- | --- | --- | --- |
| **Ribavirin** | Neutralization test in foetal rhesus kidney cells, Vero E6 cells, confirmed by plaque reduction assay | N | EC50 required for inhibition of cytopathic effect by 50% at 48 and 72 hours | (1) |
|  | Quantitative plaque reduction assay in fetal rhesus kidney, checkerboard synergy test | N | Inhibition of cytopathic effect at 48 hrs | (2) |
|  | Vero E6 cells | N | EC50 required for inhibition of cytopathic effect by 50% at 72-96 hrs | (3) |
|  | Cell types, Vero, pig kidney (PK-15), human (Caco2, CL14, and HPEK). Confluent cell cultures, infected with SARS-CoV for 1 h. | Interferon-beta + ribavirin | EC50 required for inhibition of cytopathic effect by 50% at 72 hrs | (4) |
|  | Plaque assay with confluent Vero E6 cells, microassay Vero E6 cells | N | Inhibition of cytopathic effect, plaque assay and immunoblot for specific concentrations of anti-viral | (5) |
|  | Cytopathic effects assay and plaque reduction assays in Vero E6 cells | N | Complete inhibition of cytopathic effect at 72 hrs | (6) |
| **Lopinavir or Ritonavir** | Neutralization test in foetal rhesus kidney cells, Vero E6 cells, confirmed by plaque reduction assay | N | EC50 required for inhibition of cytopathic effect by 50% at 48 and 72 hours | (1) |
|  | Quantitative plaque reduction assay in fetal rhesus kidney, checkerboard synergy test | N | Inhibition of cytopathic effect at 48 hrs | (2) |
|  | Cytopathic effects assay in Vero E6 cells, immunofluorescense assay in Vero E6 cells, RT-PCR | N | EC50 required for inhibition of cytopathic effect by 50% at 72 hrs | (7) |
| **Interferon Type I** | Neutralization test in foetal rhesus kidney cells, Vero E6 cells, confirmed by plaque reduction assay | Interferon-beta + ribavirin, interferon-alpha + ribavirin | EC50 required for inhibition of cytopathic effect by 50% at 48 and 72 hours | (2) |
|  | Confluent layers of Vero and Caco2 cells derived from human colonic tumor and treated with interferons 24 h before and after virus infection. | N | EC50 required for inhibition of cytopathic effect by 50% at 72 hrs | (8) |
|  | Pretreated, SARS-CoV inoculated Vero cells | N | Number of infected cells at different concentrations of interferon alpha | (9) |
|  | Pre and post-treated SARS-CoV-inoculated Vero E6 cells | N | EC50 required for inhibition of cytopathic effect by 50% at 24 hrs | (10) |
|  | Vero cell monolayer or confluent monolayers pre-treated with IFN's. Quantitation of RNA by TaqMan | Interferon-beta + interferon gamma | IC50 required for inhibition of cytopathic effect at 72 hrs | (11) |
|  | Plaque reduction assay with Vero cells | N | Inhibition of cytopathic effect at 24 hrs for specific concentrations of anti-viral agents | (12) |
|  | Plaque assay with confluent Vero E6 cells, microassay Vero E6 cells | N | Inhibition of cytopathic effect, plaque assay and immunoblot for specific concentrations of anti-viral | (5) |
|  | Cytopathic effects assay and plaque reduction assays in Vero E6 cells | Interferon-alpha + interferon-beta | Complete inhibition of cytopathic effect at 72 hrs | (6) |
|  | Cytopathic effects assay in Vero cells, RT-PCR and back-titration with rhesus monkey kidney cells (FRhk-4) | N | Inhibition of cytopathic effect at 36 hrs for specific concentrations of anti-viral agents | (13) |
|  | Pre and post-treated SARS-CoV-inoculated Vero E6 cells | N | IC50 required for inhibition of cytopathic effect by 50% at 24, 48 and 72 hrs | (14) |
|  | Cell types, Vero, pig kidney (PK-15), human (Caco2, CL14, and HPEK). Confluent cell cultures, infected with SARS-CoV for 1 h. | Interferon-beta + ribavirin | EC50 required for inhibition of cytopathic effect by 50% at 72 hrs | (4) |
|  | Plaque reduction assay on Vero E6 or L2 cells pre-treated with IFN, virus replication assay on Vero E6 or L2 cells. | Interferon-beta + interferon-gamma | Inhibition of cytopathic effect at 24, 48, and 72 hrs for specific concentrations of anti-viral agents | (15) |

*****N=None

**References**

1. Chen et al. In vitro susceptibility of 10 clinical isolates of SARS coronavirus to selected antiviral compounds. J Clin Virol 2004;31(1):69-75.

2. Chu et al. Role of lopinavir/ritonavir in the treatment of SARS: initial virological and clinical findings. Thorax 2004;59(3):252-6.

3. Cinatl et al. Glycyrrhizin, an active component of liquorice roots, and replication of SARS-associated coronavirus. Lancet 2003;361(9374):2045-6.

4. Morgenstern et al. Ribavirin and interferon-beta synergistically inhibit SARS-associated coronavirus replication in animal and human cell lines. Biochem Biophys Res Commun 2005;326(4):905-8.

5. Stroher et al. Severe acute respiratory syndrome-related coronavirus is inhibited by interferon- alpha. J Infect Dis 2004;189(7):1164-7.

6. Tan et al. Inhibition of SARS coronavirus infection in vitro with clinically approved antiviral drugs. Emerg Infect Dis 2004;10(4):581-6.

7. Yamamoto et al. HIV protease inhibitor nelfinavir inhibits replication of SARS-associated coronavirus. Biochem Biophys Res Commun 2004;318(3):719-25.

8. Cinatl et al. Treatment of SARS with human interferons. Lancet 2003;362(9380):293-4.

9. Haagmans et al. Pegylated interferon-alpha protects type 1 pneumocytes against SARS coronavirus infection in macaques. Nat Med 2004;10(3):290-3.

10. He et al. Potent and selective inhibition of SARS coronavirus replication by aurintricarboxylic acid. Biochem Biophys Res Commun 2004;320(4):1199-203.

11. Scagnolari C et al. Increased sensitivity of SARS-coronavirus to a combination of human type I and type II interferons. Antivir Ther 2004;9(6):1003-11.

12. Spiegel et al. The antiviral effect of interferon-beta against SARS-coronavirus is not mediated by MxA protein. J Clin Virol 2004;30(3):211-3.

13. Zheng et al. Potent inhibition of SARS-associated coronavirus (SCOV) infection and replication by type I interferons (IFN-alpha/beta) but not by type II interferon (IFN-gamma). J Interferon Cytokine Res 2004;24(7):388-90.

14. Hensley et al. Interferon-beta 1a and SARS coronavirus replication. Emerg Infect Dis 2004;10(2):317-9.

15. Sainz et al. Interferon-beta and interferon-gamma synergistically inhibit the replication of severe acute respiratory syndrome-associated coronavirus (SARS-CoV). Virology 2004;329(1):11-7.
