# Supplementary material for: SARS: Systematic Review of Treatment Effects
Source: PLoS Med. 2006 Sep 12;3(9):e343. doi: 10.1371/journal.pmed.0030343 (PMC1564166; doi:10.1371/journal.pmed.0030343)
Supplement: Supplementary file 5 [file pmed.0030343.st005.doc]

## Table S5. Results from SARS-CoV replication studies: Inhibition of SARS-CoV replication

| **Compound** | **Assay Type** | **Authors’ observations/inference** | **Conclusion** | **Reference ID** |
| --- | --- | --- | --- | --- |
| **Ribavirin** | Neutralization test in foetal rhesus kidney cells, Vero E6 cells, confirmed by plaque reduction assay | EC50 of 50-100 ug/ml in fRhK-4 cells, EC50 of >200 in Vero E6.* Ribavirin (25 ug/ml) is synergistic with interferon beta 1-a (312.5 ug/ml) or leukocytic interferon-alpha (78 ug/ml). | Evidence of some antiviral effect. Marked synergism between ribavirin and interferon | (1) |
|  | Quantitative plaque reduction assay in fetal rhesus kidney, checkerboard synergy test | Antiviral activity against SARS-CoV was demonstrated for ribavirin at concentrations of 50ug/ml at 48 hours.* | Evidence of some antiviral effect | (2) |
|  | Vero E6 cells | Ribavirin did not affect replication of SARS-CoV | No evidence of antiviral effect | (3) |
|  | Cell types, Vero, African green monkey (MA104), pig kidney (PK-15), human (Caco2, CL14, and HPEK). Confluent cell cultures, infected with SARS-CoV for 1 h. | No effect of ribavirin in Vero cells at concentrations up to 100ug/ml. However, replication was inhibited in African green monkey, porcine and human cell lines at concentrations up to 24 ug/ml*. Ribavirin with IFN-B showed a synergistic effect in human cell lines. | Evidence of some antiviral effect. Marked synergism between ribavirin and interferon | (4) |
|  | Plaque assay with confluent Vero E6 cells, microassay Vero E6 cells | SARS-CoV in Vero EG cells is not susceptible to ribavirin when treated with up to 2000ug/ml for 96 hours*.. | No evidence of antiviral effect | (5) |
|  | Cytopathic effects assay and plaque reduction assays in Vero E6 cells | Ribavirin completely inhibited CPE at 500- 5000 ug/ml at virus loads of 100-10,000 PFU per well.* | Evidence of some antiviral effect | (6) |
| **Lopinavir or Ritonavir** | Neutralization test in fetal rhesus kidney cells, Vero E6 cells, confirmed by plaque reduction assay | Lopinavir has detectable inhibitory effects against SARS-CoV at 6ug/ml.† | Evidence of some antiviral effect | (1) |
|  | Quantitative plaque reduction assay in fetal rhesus kidney, checkerboard synergy test | CPE of SARS-CoV was inhibited by lopinavir at 4ug/ml after 48 hours of incubation.† | Evidence for some antiviral effect. Marked synergism between lopinavir and ribavirin. | (2) |
|  | Cytopathic effects assay in Vero E6 cells, immunofluorescense assay in Vero E6 cells, RT-PCR | Ritonavir or lopinavir did not affect the replication of SARS-CoV | No evidence for antiviral effect | (7) |
| **Interferon Type I** | Neutralization test in foetal rhesus kidney cells, Vero E6 cells, confirmed by plaque reduction assay | Leukocytic interferon-alpha, and interferon-beta-1a, have detectable inhibitory activities. EC50 was 30U/ml and 8U/ml, respectively | Evidence for some antiviral effect. Marked synergism IFN-B or IFN-a with ribavirin. | (1) |
|  | Confluent layers of Vero and Caco2 cells derived from human colonic tumor and treated with interferons 24 h before and after virus infection. | Interferons inhibit SARS-CoV replication though interferon-beta was the most potent inhibitor. | Evidence for some antiviral effect | (8) |
|  | Pretreated, SARS-CoV inoculated Vero cells | Pegylated interferon-a significantly reduces viral replication, observed a dose dependent effect . | Evidence for some antiviral effect | (9) |
|  | Pre and post-treated SARS-CoV-inoculated Vero E6 cells | IFN-a and IFN-b had antiviral and prophylactic effects, EC50 of 500 IU/ml | Evidence for some antiviral effect | (10) |
|  | Vero cell monolayer or confluent monolayers pre-treated with IFN's. Quantitation of RNA by TaqMan | Interferon-b more effective than interferon-a or interferon-gamma. Synergism between interferon-b and interferon-gamma | Evidence for some antiviral effect. Marked synergism between IFN-B and IFN-G | (11) |
|  | Plaque reduction assay with Vero cells | Interferon-b more effective than interferon-a or interferon-gamma. | Evidence for some antiviral effect | (12) |
|  | Plaque assay with confluent Vero E6 cells, microassay Vero E6 cells | SARS CoV is inhibited in tissue culture by IFN-a-2b at concentrations >=1000 IU/mL | Evidence for some antiviral effect | (5) |
|  | Cytopathic effects assay and plaque reduction assays in Vero E6 cells | Complete inhibition was observed for interferon B-1b at 5,000 IU/ml, interferon a-n3 at 5,000 IU/ml, interferon a-n1 at 250,000 IU/ml and interferon-a at 500,000 IU/ml | Evidence for some antiviral effect | (6) |
|  | Cytopathic effects assay in Vero cells, RT-PCR and back-titration with rhesus monkey kidney cells (FRhk-4) | IFN-a and IFN-b protected cells from CPE and dose-dependently reduced SARS CoV viral RNA copies in cells. | Evidence for some antiviral effect | (13) |
|  | Pre and post-treated SARS-CoV-inoculated Vero E6 cells | IFN-B-1a exhibited potent antiviral activity when cells were pre-treated and also effective post-treated | Evidence for some antiviral effect | (14) |
|  | Cell types, Vero, pig kidney (PK-15), human (Caco2, CL14, and HPEK). Confluent cell cultures, infected with SARS-CoV for 1 h. | IFN-B combined with ribavirin reduced inhibitory concentrations of both compounds | Evidence for some antiviral effect. Marked synergism between IFN-B and ribavirin. | (4) |
|  | Plaque reduction assay on Vero E6 or L2 cells pre-treated with IFN, virus replication assay on Vero E6 or L2 cells. | In cultures treated with both IFN-B and IFN-gamma the level of inhibition was significantly greater and plaque formation was inhibited by 30 fold than either treatment alone. | Evidence for some antiviral effect. Marked synergism between IFN-B and IFN-G | (15) |

* Predicted peak plasma ribavirin concentration in man is 26 ug/mL, using standard IV regimen for treatment of hemorrhagic fever with renal syndrome (Based on Lanskin et al. (16)).

† Peak plasma lopinavir concentration in man is 9.6 +/- 4.4 ug/ml using standard oral regimen for treatment of lopinavir/ritonavir for healthy adult volunteers and in HIV-infected patients (17).

**References**

1. Chen et al. In vitro susceptibility of 10 clinical isolates of SARS coronavirus to selected antiviral compounds. J Clin Virol 2004;31(1):69-75.

2. Chu et al. Role of lopinavir/ritonavir in the treatment of SARS: initial virological and clinical findings. Thorax 2004;59(3):252-6.

3. Cinatl et al. Glycyrrhizin, an active component of liquorice roots, and replication of SARS-associated coronavirus. Lancet 2003;361(9374):2045-6.

4. Morgenstern B, Michaelis M, Baer PC, Doerr HW, Cinatl J Jr. Ribavirin and interferon-beta synergistically inhibit SARS-associated coronavirus replication in animal and human cell lines. Biochem Biophys Res Commun 2005;326(4):905-8.

5. Stroher et al. Severe acute respiratory syndrome-related coronavirus is inhibited by interferon- alpha. J Infect Dis 2004;189(7):1164-7.

6. Tan et al. Inhibition of SARS coronavirus infection in vitro with clinically approved antiviral drugs. Emerg Infect Dis 2004;10(4):581-6.

7. Yamamoto et al. HIV protease inhibitor nelfinavir inhibits replication of SARS-associated coronavirus. Biochem Biophys Res Commun 2004;318(3):719-25.

8. Cinatl et al. Treatment of SARS with human interferons. Lancet 2003;362(9380):293-4.

9. Haagmans et al. Pegylated interferon-alpha protects type 1 pneumocytes against SARS coronavirus infection in macaques. Nat Med 2004;10(3):290-3.

10. He et al. Potent and selective inhibition of SARS coronavirus replication by aurintricarboxylic acid. Biochem Biophys Res Commun 2004;320(4):1199-203.

11. Scagnolari et al. Increased sensitivity of SARS-coronavirus to a combination of human type I and type II interferons. Antivir Ther 2004;9(6):1003-11.

12. Spiegel et al. The antiviral effect of interferon-beta against SARS-coronavirus is not mediated by MxA protein. J Clin Virol 2004;30(3):211-3.

13. Zheng B, He ML, Wong KL, Lum CT, Poon LL, Peng Y et al. Potent inhibition of SARS-associated coronavirus (SCOV) infection and replication by type I interferons (IFN-alpha/beta) but not by type II interferon (IFN-gamma). J Interferon Cytokine Res 2004;24(7):388-90.

14. Hensley LE, Fritz LE, Jahrling PB, Karp CL, Huggins JW, Geisbert TW. Interferon-beta 1a and SARS coronavirus replication. Emerg Infect Dis 2004;10(2):317-9.

15. Sainz B Jr, Mossel EC, Peters CJ, Garry RF. Interferon-beta and interferon-gamma synergistically inhibit the replication of severe acute respiratory syndrome-associated coronavirus (SARS-CoV). Virology 2004;329(1):11-7.

16. Estimated from Laskin et al Ribavirin deposition in high-risk patients for acquired immunodeficiency syndrome. Clin Pharm Ther 1987; 41:546-555.

17. Abbott Laboratories. Kaletra (lopinavir/ritonavir) capsules (lopinavir/ritonavir) oral solution. 2005. Available at: http://www.fda.gov/cder/foi/label/2005/021226s016lbl.pdf Accessed on: Oct 31, 2005.
