# Supplementary material for: SARS: Systematic Review of Treatment Effects
Source: PLoS Med. 2006 Sep 12;3(9):e343. doi: 10.1371/journal.pmed.0030343 (PMC1564166; doi:10.1371/journal.pmed.0030343)
Supplement: Supplementary file 6 [file pmed.0030343.st006.doc]

**Table S6. Description of Studies within SARS Patients***

* When a study contains information about several treatments, it will appear in more than one treatment section

| **Treatment of interest** | **Country** | **Study design** | **Study N** | **Steroids?** | **Ribavirin?** | **Other treatments** | **Comparison?** | **SARS case definition?**** | **Outcomes Measured** | **Reference ID** |
| --- | --- | --- | --- | --- | --- | --- | --- | --- | --- | --- |
| **Ribavirin** | Canada | Retrospective | 110 | Some | All | - | N | U | - | (1) |
|  |  | Retrospective | 144 | Some | Some | - | N | C | Death, admission to ICU | (2) |
|  | China | RCT | 190 | Some | Some | CPAP (some) | Y^A^ | C | Death | (3) |
|  |  | Retrospective | 182 | Some | All | - | N | C | ALT, LDH | (4) |
|  |  | Retrospective | 120 | Some | All | - | N | C | Death, ventilation | (5) |
|  |  | Prospective | 13 | All | All | - | Y^B^ | C, L | Death, cytokine profiles | (6) |
|  | Hong Kong | Prospective | 138 | All | All | - | N | C, L | Death, discharge, admission to ICU | (7) |
|  |  | Prospective | 10 | Some | All | - | N | C | Death, ventilation | (8) |
|  |  | Prospective | 75 | All | All | - | N | C | Death, ventilation, ARDS | (9) |
|  |  | Prospective | 40 | All | All | - | N | C, L | Death, CT abnormalities | (10) |
|  |  | Retrospective | 323 | Some | All | - | N | L | Death, ventilation, admission to ICU | (11) |
|  |  | Retrospective | 10 | Some | All | - | N | C | Death | (12) |
|  |  | Retrospective | 109 | Some | Some | - | N | C, L | Death, ICU admission | (13) |
|  |  | Retrospective | 138 | Some | Some | - | N | C | Death, ventilation, admission to ICU | (14) |
|  |  | Retrospective | 54 | All | All | - | N | C | ALT level | (15) |
|  |  | Retrospective | 36 | All | All | - | N | C | Death | (16) |
|  |  | Retrospective | 54 | All | All | - | N | C | Death, ventilation | (17) |
|  |  | Prospective | 13 | Some | All | - | N | C | Death | (18) |
|  | Singapore | Retrospective | 229 | Some | Some | - | Y^C^ | C | Survival time, admission to ICU, ICU days | (19) |
|  |  | Retrospective | 20 | Some | Some | - | N | C | Death | (20) |
|  | Taiwan | Retrospective | 29 | Some | All | IVIG (some) | N | C | Death, ventilation, intubation | (21) |
|  |  | Retrospective | 67 | Some | Some | IVIG (some) | Y^D^ | C | Death, ventilation, ARDS | (22) |
|  |  | Retrospective | 76 | Some | Some | IVIG (some) | N | C | Death, ventilation | (23) |
|  | Vietnam | Retrospective | 62 | Some | Some | - | N | C | Death, ventilation | (24) |
| **Corticosteroids** | China | Retrospective | 67 | All | All | - | N | C | Avascular necrosis | (25) |
|  |  | RCT | 190 | Some | Some | Interferon (some), CPAP (some) | Y^A^ | C | Death | (3) |
|  |  | Retrospective | 96 | Some | Some | Interferon (some), IVIG (some) | N | C | Death | (26) |
|  |  | Prospective | 13 | All | All | - | Y^B^ | C, L | Death, cytokine profiles | (6) |
|  | Hong Kong | Retrospective | 54 | All | All | - | N | C | Death, ventilation | (17) |
|  |  | RCT | 16 | Some | All | - | Y^E^ | C | Plasma viral load | (27) |
|  |  | Case-control | 45 | All | - | - | Y^F^ | U | Dose of steroid | (28) |
|  |  | Retrospective | 57 | Some | - | - |  | L | CT abnormalities | (29) |
|  |  | Prospective | 40 | All | All | - | N | C, L | Death, CT abnormalities | (10) |
|  |  | Retrospective | 72 | Some | All | - | Y^G^ | C | Death, ventilation, admission to ICU, oxygen requirement | (30) |
|  |  | Prospective | 88 | All | All |  | N | L | Death, discharge | (31) |
|  |  | Prospective | 138 | All | All |  | N | C, L | Death, discharge, admission to ICU | (7) |
|  |  | Retrospective | 36 | All | All | - | N | C | Death | (16) |
|  | Taiwan | Retrospective | 67 | Some | Some | IVIG (some) | Y^D^ | C | Death, ventilation, ARDS | (22) |
|  |  | Retrospective | 76 | Some | Some | IVIG (some) | N | C | Death, ventilation | (23) |
| **Lopinavir and**  **Ritonavir** | Hong Kong | Retrospective | 1052 | All | All |  | Y^H^ | C | Death, intubation | (32) |
|  |  | Retrospective | 152 | All | All |  | Y^H^ | C | Death, ARDS | (33) |
| **Interferon-alpha** | Canada | Retrospective | 22 | All | - |  | Y^I^ | C | Death, ventilation, admission to ICU | (34) |
|  | China | RCT | 190 | Some | Some | CPAP (some) | Y^A^ | C | Death | (3) |
| **Immunoglobulin** | Hong Kong | Retrospective | 12 | All | All | - | N | C, L | Discharge | (35) |
|  | Taiwan | Retrospective | 67 | Some | Some | IVIG (some) | Y^D^ | C | Death, ventilation, ARDS | (22) |
|  |  | Retrospective | 76 | Some | Some | IVIG (some) | N | C | Death, ventilation | (23) |
| **Convalescent plasma** | Hong Kong | Retrospective | 40 | All | All | - | Y^J^ | C | Death | (36) |
|  | Hong Kong | Retrospective | 80 | Some | Some | - | N | C | Death, discharge | (37) |

**C=Clinical and epidemiological criteria established by WHO, CDC or an equivalent criteria was met. L=laboratory evidence of SARS-CoV was obtained serologically or by reverse transcriptase polymerase chain reaction. U=unclear.

A. CPAP + ribavirin vs. interferon-alpha vs. interferon-alpha plus early steroids if needed vs. interferon-alpha plus early steroids if needed plus CPAP

B. Cytokine levels in SARS patients were compared with data from 60 healthy Chinese subjects of similar age and sex distribution.

C. Ribavirin vs. without ribavirin

D. Development of ARDS vs. no ARDS

E. Steroids vs. placebo

F. Diagnosis of psychosis vs. No psychosis

G. Pulse steroids vs. non-pulse steroid

H. Early lopinavir/ritonavir vs. late lopinavir/ritonavir and lopinavir/ritonavir vs. standard treatment

I. Interferon-a vs without interferon-a

J. Convalescent plasma vs. steroids for rescue treatment

**References**

1. Knowles et al. Common adverse events associated with the use of ribavirin for severe acute respiratory syndrome in Canada. Clin Infect Dis 2003;37(8):1139-42.

2.	Booth et al. Clinical features and short-term outcomes of 144 patients with SARS in the greater Toronto area. JAMA 2003;289(21):2801-9.

3.	Zhao et al. Description and clinical treatment of an early outbreak of severe acute respiratory syndrome (SARS) in Guangzhou, PR China. 2003;52(8):715-20.

4.	Cui et al. Serum hepatic enzyme manifestations in patients with severe acute respiratory syndrome: Retrospective analysis. World Journal of Gastroenterology 2004;10(11):1652-5.

5.	Han et al. Noninvasive positive pressure ventilation treatment for acute respiratory failure in SARS. Sleep and Breathing 2004;8(2):97-106.

6.	Jones et al. Prolonged disturbances of in vitro cytokine production in patients with severe acute respiratory syndrome (SARS) treated with ribavirin and steroids. Clin Exp Immunol 2004;135(3):467-73.

7.	Sung et al. Severe acute respiratory syndrome: report of treatment and outcome after a major outbreak. Thorax 2004;59(5):414-20.

8.	Hon et al. Clinical presentations and outcome of severe acute respiratory syndrome in children. Lancet 2003;361(9370):1701-3.

9.	Peiris et al. Clinical progression and viral load in a community outbreak of coronavirus-associated SARS pneumonia: a prospective study. Lancet 2003;361(9371):1767-72.

10.	Ooi et al. Severe Acute Respiratory Syndrome: Radiographic Evaluation and Clinical Outcome Measures. Radiology 2003;229(2):500-6.

11.	Tsui et al. Severe acute respiratory syndrome: Clinical outcome and prognostic correlates. 2003;9(9):1064-9.

12.	Tsang et al. A cluster of cases of severe acute respiratory syndrome in Hong Kong. N Engl J Med 2003;348(20):1977-85.

13.	Chan et al. Serum LD1 isoenzyme and blood lymphocyte subsets as prognostic indicators for severe acute respiratory syndrome. Journal of Internal Medicine 2004;255(4):512-8.

14.	Lee et al. A major outbreak of severe acute respiratory syndrome in Hong Kong. New England Journal of Medicine 2003;348(20):1986-94.

15.	Wong et al. Temporal Patterns of Hepatic Dysfunction and Disease Severity in Patients with SARS (4). JAMA 2003;290(20):2663-5.

16.	Tiwari et al. Severe acute respiratory syndrome (SARS) in Hong Kong: patients' experiences. Nurs Outlook 2003;51(5):212-9.

17.	Gomersall et al. Short-term outcome of critically ill patients with severe acute respiratory syndrome. Intensive Care Med 2004;30(3):381-7.

18.	Cheng et al. Ribavirin for SARS in children. Clin Pediatr (Phila) 2004;43(2):193-6.

19.	Leong et al. Investigational use of ribavirin in the treatment of severe acute respiratory syndrome, Singapore, 2003. Trop Med Int Health 2004;9(8):923-7.

20.	Hsu et al. Severe acute respiratory syndrome (SARS) in Singapore: Clinical features of index patient and initial contacts. 2003;9(6):713-7.

21.	Jang et al. Severe acute respiratory syndrome in Taiwan: analysis of epidemiological characteristics in 29 cases. J Infect 2004;48(1):23-31.

22.	Chen et al. Clinical features and outcomes of severe acute respiratory syndrome and predictive factors for acute respiratory distress syndrome. Journal of the Chinese Medical Association 2005;68(1):4-10.

23.	Wang et al. Clinical Manifestations, Laboratory Findings, and Treatment Outcomes of SARS Patients. 2004;10(5):818-24.

24.	Vu et al. Clinical Description of a Completed Outbreak of SARS in Vietnam February-May 2003. Emerging Infectious Diseases 2004;10(2):334-8.

25.	Hong N, Du XK. Avascular necrosis of bone in severe acute respiratory syndrome. Clin Radiol 2004;59(7):602-8.

26.	Wu et al. A hospital outbreak of severe acute respiratory syndrome in Guangzhou, China. Chin Med J (Engl) 2003;116(6):811-8.

27.	Lee et al. Effects of early corticosteroid treatment on plasma SARS-associated Coronavirus RNA concentrations in adult patients. J Clin Virol 2004;31(4):304-9.

28.	Lee et al. Factors associated with psychosis among patients with severe acute respiratory syndrome: A case-control study. Clinical Infectious Diseases 2004;39(8):1247-9.

29.	Ng et al. Six month radiological and physiological outcomes in severe acute respiratory syndrome (SARS) survivors. Thorax 2004;59(10):889-91.

30.	Ho et al. High-dose pulse versus nonpulse corticosteroid regimens in severe acute respiratory syndrome. Am J Respir Crit Care Med 2003;168(12):1449-56.

31.	Lau et al. Outcome of coronavirus-associated severe acute respiratory syndrome using a standard treatment protocol. Respirology 2004;9(2):173-83.

32.	Chan et al. Treatment of severe acute respiratory syndrome with lopinavir/ritonavir: a multicentre retrospective matched cohort study. Hong Kong Med J 2003;9(6):399-406.

33.	Chu et al. Role of lopinavir/ritonavir in the treatment of SARS: initial virological and clinical findings. Thorax 2004;59(3):252-6.

34.	Loutfy et al. Interferon alfacon-1 plus corticosteroids in severe acute respiratory syndrome: a preliminary study. JAMA 2003;290(24):3222-8.

35.	Ho et al. Pentaglobin in steroid-resistant severe acute respiratory syndrome. Int J Tuberc Lung Dis 2004;8(10):1173-9.

36.	Soo et al. Retrospective comparison of convalescent plasma with continuing high-dose methylprednisolone treatment in SARS patients. Clin Microbiol Infect 2004;10(7):676-8.

37.	Cheng et al. Use of convalescent plasma therapy in SARS patients in Hong Kong. Eur J Clin Microbiol Infect Dis 2005;24(1):44-6.
