# Supplementary material for: SARS: Systematic Review of Treatment Effects
Source: PLoS Med. 2006 Sep 12;3(9):e343. doi: 10.1371/journal.pmed.0030343 (PMC1564166; doi:10.1371/journal.pmed.0030343)
Supplement: Supplementary file 7 [file pmed.0030343.st007.doc]

**Table S7. Results of Treatment within SARS Patients (English literature)**

| **Treatment of interest** | **Dose** | **Authors’ observations/inference** | **Critical appraisal** | **Conclusion** | **Reference ID** |
| --- | --- | --- | --- | --- | --- |
| **Ribavirin** | Not available | Haemolytic anaemia: 67/110. Hypomagnesaemia: 35/76. Authors conclude “adverse effects occur frequently and have a dose-response relationship with ribavirin.” | Haemoglobin levels at admission and after treatment were compared. Frequent side effects such as haemolytic anaemia and hypomagnesaemia were noted. Not enough evidence to ascertain causality due to lack of control group and the fact that some patients were given steroids. | Possible harm | (1) |
|  | 2g IV then 1g IV every 6 h for 4 days | Death: 8/144; haemolytic anaemia: 71/126. Authors conclude “ribavirin is temporarily associated with significant toxicity.” | Haemoglobin levels at admission and after treatment were compared. Haemolysis and fall in haemoglobin were common. Not enough evidence to ascertain causality due to lack of control group and the fact that some patients were given steroids. | Possible harm | (2) |
|  | 0.4-0.6g/day^-1^ IV | Death in ribavirin and CPAP group :2/40  Death in no ribavirin group: 9/90.  Authors conclude “no advantage seen” | Comparison made between patients given both ribavrin and CPAP to those treated with other than ribavirin. Treatment regimens appear to be neither standardized nor randomized. Possible confounding by the epidemic. Cannot distinguish effect of ribavirin from other treatments. | Inconclusive | (3) |
|  | Not available | Abnormal liver enzyme activity after treatment in 70%. Liver enzyme activities were not correlated with duration of treatment. Authors conclude “SARS might affect the liver and induce damage in the course of infection” | Liver function outcomes before and after treatment were compared. Correlation of ribavirin treatment with abnormal liver enzyme activities not clear. Subset of patients treated in a different hospital may have differed in illness severity. Causality unclear as no appropriate control group available and some patients were treated with steroids. | Inconclusive | (4) |
|  | Not available | Death: 8/120. | All patients received ribavirin, some received steroids; without a control group treated differently, it is not clear if ribavirin was of any benefit. | Inconclusive | (5) |
|  | 400 mg IV or 2.4 g then 1.2g oral, 3 times daily | Haemolytic anemia: 49/138.  Response to ribavirin and low dose steroid treatment: 25/107 | Haemoglobin levels at baseline and 2 wks after treatment were compared. Haemolytic anaemia and hyperglycaemia were common. Causality unclear due to lack of a control group and co-treatment with steroids. | Possible harm | (6) |
|  | 40 mg/kg oral daily | Short-term use of ribavirin was well tolerated and had no major short-term adverse effects such as severe haemolytic anaemia: 10/10 paediatric patients | Article type is a research letter. Treatment effect of ribavirin cannot be established due to lack of a control group. Good outcome may be due to mild SARS illness in children. | Inconclusive | (7) |
|  | 8 mg/kg IV every 8 h for 14 days | Afebrile within 48 hours of ribavirin and steroids: 74/75. Fever recurred in 64/75 of patients at a mean of 8.9 days.  Death: 5/75 | Anaemia occurred in 8% but unclear if due to ribavirin, illness or co-treatment. Unclear if ribavirin of any benefit due to lack of control group. | Inconclusive | (8) |
|  | 8 mg/kg IV 3 times/day or 1.2 g oral 3 times/day | Good response: 17/40, Fair response: 9/40 Poor response : 14/40. Three distinct categories of patients who differ in treatment response and severity of disease at presentation. | SARS confirmed by serology. Treatment response determined at 5 days post treatment. Pre-determined outcome categories were “good, fair, poor.” Unclear if ribavirin of any benefit since all received steroids and lack of control group. | Inconclusive | (9) |
|  | 8 mg/kg IV every 8 h. If extensive pneumonitis: loading dose of 33 mg/kg followed by 20mg/kg every 8 h. or oral equivalent. | Illness progression: 208/323. Many patients progressed to pneumonitis despite ribavirin and steroid combination therapy.  Death: 26/323 | SARS confirmed by serology. Authors found patients who sought treatment early and received ribavirin and steroid combination therapy did not benefit compared to those who sought treatment later. Early treatment-seekers were given antivirals later in illness. Unclear if ribavirin of any benefit due to lack of a control group. | Inconclusive | (10) |
|  | 8mg/kg IV every 8 h. one patient received oral 1.2g every 8 h. | Empirical treatment with a combination of high-dose corticosteroid and ribavirin coincided with clinical improvement. | No information on how many patients improved following ribavirin treatment. Benefit or harm cannot be determined due to very small sample size of 10. | Inconclusive | (11) |
|  | 400 mg IV every 8 h for 14 days. Or oral: 1.2 g 3 times daily after leading dose of 2.4g. | Death: 42/109  Raised serum LD1 level was a predictor of death in a multiple ROC curve comparison of prognostic indicators. A haemolytic effect of ribavirin cannot be excluded as a possible cause. | High mortality in this cohort. Unclear if ribavirin of any benefit or harm due to lack of a control group. | Inconclusive | (12) |
|  | 1.2 g oral 3 times/day. If worsened then 400 mg IV every 8 hours. | Death: 5/138  Respiratory failure: 32/138 | Unclear if ribavirin of any benefit or harm due to lack of a control group. | Inconclusive | (13) |
|  | Not available | Liver dysfunction: 29.6% before ribavirin and 75.9% during treatment. Liver dysfunction is a distinct abnormality in patients with SARS. | Study compared biochemical levels in SARS patients pre- and post- treatment. Also compared levels to patients without SARS. Data regarding dosage of ribavirin is not shown. Ribavirin may have caused liver dysfunction but difficult to establish causality due to lack of control group and co-treatment with steroids. | Possible harm | (14) |
|  | IV 8mg/kg tid, oral: 1.2g tid | No outcomes reported | Data is very poor quality because outcome is the current state of patient on a single day of chart review. No control group. | Inconclusive | (15) |
|  | 8 mg/kg IV every 8 hours for 7-10 days followed by 4 mg/kg enterally for 11-14 days | Death: 14/54. Mortality amongst critically ill patients with SARS is high. | Mortality is expected to be high because cohort consists of ICU patients. Lack of a control group and co-treatment with steroids prevents a conclusion on treatment effect. | Inconclusive | (16) |
|  | 1.2g tid oral; or 400 mg IV every 8 h | Death in ribavirin group: 10.3%;  Death in control group: 12.3%. Hazard ratio indicated no significant difference. Authors acknowledge limitations but suggest that ribavirin confers no benefit. | Cox regression analysis used to compare survival time between those given ribavirin and those not. Adjusted for predictors of poor prognosis. Treatment allocation was not randomised and bias may have affected the results. Groups were treated at different times of the epidemic, ribavirin group treated earlier. | Inconclusive | (17) |
|  | 2.0g oral then 100-1200 mg daily; or IV 400mg every 8h | Death: 4/29. Anemia:14/29 patients. “No obvious therapeutic response” to ribavirin. | No comparisons or tests of significance used. Results are anecdotal. Lack of a control group and patients also treated with steroids and immunoglobulin. | Inconclusive | (18) |
|  | Not available | Death: 6/62. After 18 patients received treatment for average of 9 days, authors note “no clinically beneficial effect on the course of illness” | Findings in regard to treatment are non-specific. No protocol for the timing or dosage of steroids. Conclusions on treatment cannot be made due to lack of a control group. | Inconclusive | (19) |
|  | Not available | ARDS was associated with significant mortality despite aggressive therapy. Lymphopenia was common and probably because of ribavirin therapy. | Study looked for associations with ARDS. Not possible to determine specific treatment effect since many different treatments were given together. Treatment determined by physicians and not defined by specific criteria. | Inconclusive | (20) |
|  | 8 mg/kg 3 times/day IV for 3 days then 1200 mg 3 times/day orally for 10 days. | Death: 2/13. Ribavirin was of limited value in reducing proinflammatory cytokines secondarily to neutralising the infecting virus. | This study compared cytokine levels in SARS patients with healthy controls of similar age and sex. | Inconclusive | (21) |
|  | 40-60 mg/kg/day oral | Death 0/13. All patients tolerated high dose ribavirin well without major side effects during a 6-week follow-up period. Results may not be directly applicable to adults. | Unclear if ribavirin is of any benefit due to lack of a control group and small sample size. | Inconclusive | (22) |
|  | 20 mg/kg 3 times/day. | Death 3/20. No clinical benefit noted with ribavirin. Of note, Treatment was started late in course of illness, 10-14 days after symptoms. | Very early report on the index case and initial contacts in Singapore. Outcomes not specific to treatment and lack of control group prohibits conclusion on treatment effectiveness. | Inconclusive | (23) |
|  | 2,000 mg followed by 1,200 mg/day if body weight >75kg, or 1,000 mg/day if body weight <75 kg. Treatment lasted 10 days. | Death 15/76. 23.1% of those who received steroids had rebound or persistance of fever after initial use. 18 patients developed serious complications, most commonly infections. | Lack of control group, underlying conditions in several patients and combination of treatment prevents conclusion on treatment effectiveness. | Inconclusive | (24) |
| **Corticosteroids** | IV MP 80-800 mg daily for 3-20 days | Avascular necrosis: 28/65 of those with large joint pain. Unknown whether the virus itself or with steroid use could lead to AVN | No information on denominator treated with steroids. Study includes only those who had large joint pain. | Inconclusive | (25) |
|  | MP 80-160 mg/day for 2-3 days or high-dose MP 160-1000 mg/day for 5-14 days | Death: 0/60. Early use of high-dose methylprednisolone in combination with a quinolone plus azithromycin gave best outcome. | Treatment regimens appear to be neither standardized nor randomized. Possible confounding by the epidemic. | Inconclusive | (3) |
|  | MP, initial mean dose 67.3 mg/d, maximal dose 82.4 mg/d. | Death: 1/96. Effects of treatments cannot be differentiated. 2/3 of cohort received methylprednisolone and appeared to do well. | Not clear which patients received which treatments and how this influenced outcome. | Inconclusive | (26) |
|  | Low-dose corticosteroids with pulsed MP 500mg up to a total 3-5 g. | Death: 14/54. Mortality amongst critically ill patients with SARS is high. Lower steroid dose was one of several variables associated with poor outcome. No proof of beneficial effect. | Confounding of indicators used to predict poor outcome is likely. Cannot attribute good outcome to higher steroid dose. | Inconclusive | (16) |
|  | Early IV hydrocortisone 100 mg every 8 hours | Early (<7 days after fever) corticosteroid treatment delayed viral clearance and was associated with higher plasma viral load (SARS-CoV): | Prospective, randomized double blind trial gives weak evidence of delayed viral clearance with hydrocortisone treatment during the first week of illness in a very small sample size. | Possible harm | (27) |
|  | NA | Patients with psychosis received higher cumulative doses of steroids than steroid-treated controls without psychosis. | Psychotic diagnosis made by clinical assessment, not standardized interview. In this case control study patients with psychosis had higher rates of family history of psychiatric illness. Age and sex-matched control group of SARS patients without psychosis. | Possible harm | (28) |
|  | No information | Lung function abnormalities 6 months after illness: 43/57 Use of pulse corticosteroids was associated with CT abnormalities. Significant abnormalities exist in many patients 6 months after illness | Cannot determine if the association between pulse corticosteroids and CT abnormalities is causal without a control group not treated with corticosteroids. | Inconclusive | (29) |
|  | Hydrocortisone 2 mg/kg every 6 hours for 3 days then oral prednisolone, 2mg/ kg. IV MP, 2-3mg/kg daily for 3days or IV pulsed MP 500 mg for 3-5 days | Good response: 17/40. fair: 9/40. poor: 14/40. | Treatment response determined at 5 days post treatment. Pre-determined outcome categories were “good, fair, poor.” Unclear if steroids of any benefit due to lack of control group. | Inconclusive | (9) |
|  | Pulsed-regimen, MP ≥500 mg/d  Non-pulsed regimen: MP <500 mg/d | Death in high-dose, pulsed regimen: 1/17. Death among non-pulsed regimen: 3/55. The pulse steroid group had less oxygen requirement and better outcome. Patients treated with pulse steroids had less hyperglycemia (p=.004) | Patients treated with non-pulsed steroids were compared to those treated with pulsed. Since treatments were not randomized, could be confounded by baseline clinical characteristics and illness severity. | Inconclusive | (30) |
|  | MP 3mg/kg daily IV for 5 days. Then prednisolone 1mg/kg daily orally for 5 days. Pulsed MP rescue: 500mg IV twice daily for 2 days. | Death: 3/88. Hyperglycemia after steroid treatment occurred in 58%. High dose corticosteroids may be required at the start of treatment to achieve disease control. | Authors discuss the timing of steroid in their protocol is targeted to onset of immune lung damage. No analysis of treatment timing can be made within the patient cohort. Relatively low mortality cannot be attributed solely to treatment regimen. | Inconclusive | (31) |
|  | Prednisolone on day 3-4 of illness: 0.5-1mg/kg. If dyspnea then hydrocortisione 100mg every 8 h. Pulse MP rescue: 0.5 g IV for 3 days) | Death: 13/158  81.9% failed to improve on ribavirin and low dose steroids. 95/107 treated with high dose MP had some favourable response. Use of high dose MP during clinical progression phase may be effective but clinical trials needed. | High percentage of patients improved with high dose MP, during phase 2 of illness. However, improvement cannot be clearly attributed to this treatment regimen. | Inconclusive | (6) |
|  | MP IV 500-1000mg/daily some followed by oral prednisolone 80-140mg daily | No outcomes reported | Data is very poor quality because outcome is the current state of patient on a single day of chart review. No control group. | Inconclusive | (15) |
|  | Not available | ARDS was associated with significant mortality in SARS patients despite aggressive therapy. | Study looked for associations with ARDS. Not possible to determine specific treatment effect since many different treatments were given together. Treatment determined by physicians and not defined by specific criteria. | Inconclusive | (20) |
|  | IV hydrocortisone or methylprednisolone for 1-19 days starting 1-7 days after admission, followed by oral prednisolone for 30-36 days if no recovery. | Death: 2/13. Prolonged high doses used failed to reduce rapid production of inflammatory cytoklines. | This study compared cytokine levels in SARS patients with healthy controls of similar age and sex. | Inconclusive | (21) |
|  | Methylprednisolone 2 mg/kg/day for 5 days was usually administered in the second week as necessary. Pulse dosage of 500 mg/day for 3 days if disease progressed. | Death rate 19.7%. 23.1% of those who received steroids had rebound or persistance of fever after initial use. 18 patients developed serious complications, most commonly infections. | Lack of control group, underlying conditions in several patients and combination of treatment prevents conclusion on treatment effectiveness. | Inconclusive | (24) |
| **Lopinavir and Ritonavir** | Lopinavir 400 mg/ritonavir 100mg orally every 12 hours | Death rate in LPV/r initial treatment group: 2.3% Death rate control: 15.6%. Death rate in LPV/r rescue group: 12.9% Death rate control: 14%. The addition of LPV/r to a standard treatment protocol as an initial treatment appears to be associated with reduction mortality rate, reduction in intubation and reduction of rescue steroids. | Large study of 1052 patients who were not randomly assigned to treatment and control groups, however patients were matched on prognostic factors. Physicians assigned treatment regimen and may have treated more severe patients with the LPV/r rescue protocol. | Inconclusive | (32) |
|  | Lopinavir 400 mg/ritonavir 100 mg orally every 12 hours | Composite outcome up to 21 days of severe hypoxaemia or death: 1/41 for LPV/r v 32/111 for control. LPV/r was associated with better outcome in a multivariate analysis. 29/41 LPV/r treated had a >2g/dl fall in haemoglobin. Patients with LPV/r as an initial treatment seemed to run a milder disease and had a reduction in viral load | A historical control group was used. There were differences in treatment and control group in terms of sex, platelet counts, LDH level. Positive effect of L/R could have been exaggerated by worse than expected outcome in controls. | Inconclusive | (33) |
| **Interferon-alpha** | 9ug/d for 2 days then increased to 15 ug/d if no clinical response | Death: 0/9 in interferon-a +steroid group. Death: 1/13 in steroid only group. IFN-a was well tolerated. Evidence that IFN-a may have more rapid resolution of lung x-ray and better oxygen saturation levels than corticosteroids alone. | Control group was patients admitted at the same time and treated with corticosteroids only or those who declined interferon treatment. Lack of randomization and small sample size makes effect of treatment difficult to conclude. | Inconclusive | (34) |
|  | IM 3,000,000 U/day | The combined use of interferon and large dose of immunoglobulin had no obvious effect. | Patients in each group were given CPAP inconsistently. Lack of clear-cut use of any treatment in any group makes it difficult to find an effect. | Inconclusive | (3) |
| **Immunoglobulin or Convalescent Plasma** | N/A | There was an improvement in radiographic score 5, 6, and 7 days after treatment with IVIg, compared to day 1. Patients recovered after treatment. | IVIg was given after deterioration on corticosteroid and ribavirin treatment. Cannot tell if improvement was attributed to IVIg. | Inconclusive | (35) |
|  | N/A | Death rate: 6.3% early group v 21.9% later group. No adverse reactions seen. Better outcome if convalescent plasma given before day 14 of illness and among those who were still sero-negative for SARS-CoV. | Patients received plasma at different time during illness. Without a control group, cannot be sure that improvement is due to convalescent plasma. | Inconclusive | (36) |
|  | N/A | Death: 0/19 vs 5/21 in control. Convalescent plasma associated with a more favourable outcome in SARS patients who deteriorated despite ribavirin and high dose MP. | Either plasma or steroids given at discretion of physician and based on availability of plasma. Steroid group had more co-morbidities. Cannot attribute improvement to treatment alone. | Inconclusive | (37) |
|  | Intravenous immunoglobulin | ARDS was associated with significant mortality in SARS patients despite aggressive therapy with ribavirin, steroids and IVIG. | Study looked for associations with ARDS. Not possible to determine specific treatment effect since many different treatments were given together. Treatment determined by physicians and not defined by specific criteria. | Inconclusive | (20) |
|  | Intravenous immunoglobulin 1g/kg/day for 2 days | Death rate 19.7%. Authors suggest that IVIG appeared effective in controlling leukopenia and thrombocytopenia. | Lack of control group, underlying conditions in several patients and combination of treatment prevents conclusion on treatment effectiveness. | Inconclusive | (24) |

**References**

1.	Knowles et al. Common adverse events associated with the use of ribavirin for severe acute respiratory syndrome in Canada. Clin Infect Dis 2003;37(8):1139-42.

2.	Booth et al. Clinical features and short-term outcomes of 144 patients with SARS in the greater Toronto area. JAMA 2003;289(21):2801-9.

3.	Zhao et al. Description and clinical treatment of an early outbreak of severe acute respiratory syndrome (SARS) in Guangzhou, PR China. 2003;52(8):715-20.

4.	Cui et al. Serum hepatic enzyme manifestations in patients with severe acute respiratory syndrome: Retrospective analysis. World Journal of Gastroenterology 2004;10(11):1652-5.

5.	Han et al. Noninvasive positive pressure ventilation treatment for acute respiratory failure in SARS. Sleep and Breathing 2004;8(2):97-106.

6.	Sung et al. Severe acute respiratory syndrome: report of treatment and outcome after a major outbreak. Thorax 2004;59(5):414-20.

7.	Hon et al. Clinical presentations and outcome of severe acute respiratory syndrome in children. Lancet 2003;361(9370):1701-3.

8.	Peiris et al. Clinical progression and viral load in a community outbreak of coronavirus-associated SARS pneumonia: a prospective study. Lancet 2003;361(9371):1767-72.

9.	Ooi et al. Severe Acute Respiratory Syndrome: Radiographic Evaluation and Clinical Outcome Measures. Radiology 2003;229(2):500-6.

10.	Tsui et al. Severe acute respiratory syndrome: Clinical outcome and prognostic correlates. 2003;9(9):1064-9.

11.	Tsang et al. A cluster of cases of severe acute respiratory syndrome in Hong Kong. N Engl J Med 2003;348(20):1977-85.

12.	Chan et al. Serum LD1 isoenzyme and blood lymphocyte subsets as prognostic indicators for severe acute respiratory syndrome. Journal of Internal Medicine 2004;255(4):512-8.

13.	Lee et al. A major outbreak of severe acute respiratory syndrome in Hong Kong. New England Journal of Medicine 2003;348(20):1986-94.

14.	Wong et al. Temporal Patterns of Hepatic Dysfunction and Disease Severity in Patients with SARS (4). JAMA 2003;290(20):2663-5.

15.	Tiwari et al. Severe acute respiratory syndrome (SARS) in Hong Kong: patients' experiences. Nurs Outlook 2003;51(5):212-9.

16.	Gomersall et al. Short-term outcome of critically ill patients with severe acute respiratory syndrome. Intensive Care Med 2004;30(3):381-7.

17.	Leong et al. Investigational use of ribavirin in the treatment of severe acute respiratory syndrome, Singapore, 2003. Trop Med Int Health 2004;9(8):923-7.

18.	Jang et al. Severe acute respiratory syndrome in Taiwan: analysis of epidemiological characteristics in 29 cases. J Infect 2004;48(1):23-31.

19.	Vu et al. Clinical Description of a Completed Outbreak of SARS in Vietnam February-May 2003. Emerging Infectious Diseases 2004;10(2):334-8.

20.	Chen et al. Clinical features and outcomes of severe acute respiratory syndrome and predictive factors for acute respiratory distress syndrome. Journal of the Chinese Medical Association 2005;68(1):4-10.

21.	Jones et al. Prolonged disturbances of in vitro cytokine production in patients with severe acute respiratory syndrome (SARS) treated with ribavirin and steroids. Clin Exp Immunol 2004;135(3):467-73.

22.	Cheng et al. Ribavirin for SARS in children. Clin Pediatr (Phila) 2004;43(2):193-6.

23.	Hsu et al. Severe acute respiratory syndrome (SARS) in Singapore: Clinical features of index patient and initial contacts. 2003;9(6):713-7.

24.	Wang et al. Clinical Manifestations, Laboratory Findings, and Treatment Outcomes of SARS Patients. 2004;10(5):818-24.

25.	Hong N, Du XK. Avascular necrosis of bone in severe acute respiratory syndrome. Clin Radiol 2004;59(7):602-8.

26.	Wu et al. A hospital outbreak of severe acute respiratory syndrome in Guangzhou, China. Chin Med J (Engl) 2003;116(6):811-8.

27.	Lee et al. Effects of early corticosteroid treatment on plasma SARS-associated Coronavirus RNA concentrations in adult patients. J Clin Virol 2004;31(4):304-9.

28.	Lee et al. Factors associated with psychosis among patients with severe acute respiratory syndrome: A case-control study. Clinical Infectious Diseases 2004;39(8):1247-9.

29.	Ng et al. Six month radiological and physiological outcomes in severe acute respiratory syndrome (SARS) survivors. Thorax 2004;59(10):889-91.

30.	Ho et al. High-dose pulse versus nonpulse corticosteroid regimens in severe acute respiratory syndrome. Am J Respir Crit Care Med 2003;168(12):1449-56.

31.	Lau et al. Outcome of coronavirus-associated severe acute respiratory syndrome using a standard treatment protocol. Respirology 2004;9(2):173-83.

32.	Chan et al. Treatment of severe acute respiratory syndrome with lopinavir/ritonavir: a multicentre retrospective matched cohort study. Hong Kong Med J 2003;9(6):399-406.

33.	Chu et al. Role of lopinavir/ritonavir in the treatment of SARS: initial virological and clinical findings. Thorax 2004;59(3):252-6.

34.	Loutfy et al. Interferon alfacon-1 plus corticosteroids in severe acute respiratory syndrome: a preliminary study. JAMA 2003;290(24):3222-8.

35.	Ho et al. Pentaglobin in steroid-resistant severe acute respiratory syndrome. Int J Tuberc Lung Dis 2004;8(10):1173-9.

36.	Cheng et al. Use of convalescent plasma therapy in SARS patients in Hong Kong. Eur J Clin Microbiol Infect Dis 2005;24(1):44-6.

37.	Soo et al. Retrospective comparison of convalescent plasma with continuing high-dose methylprednisolone treatment in SARS patients. Clin Microbiol Infect 2004;10(7):676-8.
