# Supplementary material for: SARS: Systematic Review of Treatment Effects
Source: PLoS Med. 2006 Sep 12;3(9):e343. doi: 10.1371/journal.pmed.0030343 (PMC1564166; doi:10.1371/journal.pmed.0030343)
Supplement: Supplementary file 8 [file pmed.0030343.st008.doc]

**Table S8**. Description of Studies of SARS patients (Chinese Literature)

| **Treatment of interest** | **Study design** | **Study N** | **Steroids?** | **Ribavirin?** | **Other treatments** | **Comparison?** | **SARS case definition? **** | **Outcomes Measured** | **Reference ID** |
| --- | --- | --- | --- | --- | --- | --- | --- | --- | --- |
| **Ribavirin** | Retrospective | 43 | All | All | Interferon-a, IgG | N | C | Death | (1) |
|  | Retrospective | 29 | Some | Some | Convalescent plasma | N | C | Death, clinical outcomes | (2) |
|  | Retrospective | 96 | Some | Some | Lopinavir/ritonavir, interferon-a, convalescent plasma | N | U | Death, clinical outcomes | (3) |
|  | Retrospective | 41 | Some | All | - | N | C | Death, discharge, rescue steroid use | (4) |
|  | Retrospective | 18 | All | All | IgG | N | C, L | Clinical outcomes | (5) |
|  | Retrospective | 77 | All | All | - | N | C | Death, discharge | (6) |
| **Corticosteroids** | Retrospective | 30 | All | ? | Thymosin | N | C | Arthralgia | (7) |
|  | Retrospective | 133 | Some | All | - | Y^A^ | C | T-lymphocyte counts | (8) |
|  | Retrospective | 37 | All | None | Chinese medicine | N | U | Death | (9) |
|  | Retrospective | 132 | Some | None | - | N | U | Corticoid-induced diabetes | (10) |
|  | Retrospective | 38 | All | None | IgG, antiviral | N | C | Death, admission to ICU | (11) |
|  | Retrospective | 43 | Some | All | Thymus | N | U | Clinical outcomes | (12) |
|  | Retrospective | 40 | All | All | Thymus | N | C, L | Avascular necrosis | (13) |
|  | Retrospective | 1291 | Some | Some | - | Y^B^ | C | Death | (14) |
|  | Retrospective | 45 | All | None | - | N | C | Clinical outcomes | (15) |
|  | Prospective | 106 | Some | All | - | N | C | Death, clinical outcomes | (16) |
|  | Retrospective | 96 | Some | Some | Lopinavir/ritonavir, interferon-a, convalescent plasma | N | U | Death, clinical outcomes | (3) |
|  | Retrospective | 41 | Some | All | - | N | C | Death, discharge, rescue steroid use | (4) |
|  | Retrospective | 18 | All | All | IgG | N | C, L | Clinical outcomes | (5) |
|  | Retrospective | 77 | Some | None | - | N | C | Death, clinical outcome | (17) |
| **Interferon type I** | Retrospective | 96 | Some | Some | Lopinavir/ritonavir, interferon-a, convalescent plasma | N | U | Death, clinical outcomes | (3) |
| **Convalescent plasma/**  **Immunoglobulin** | Retrospective | 96 | Some | Some | Lopinavir/ritonavir, interferon-a, ribavirin | N | U | Death, clinical outcomes | (3) |
|  | Retrospective | 18 | All | All | Ribavirin | N | C, L | Clinical outcomes | (5) |

**C=Clinical and epidemiological criteria established by WHO, CDC or an equivalent criteria was met. L=laboratory evidence of SARS-CoV was obtained serologically or by reverse transcriptase polymerase chain reaction. U=unclear.

A. Steroids (N=99) of these, high dose methylprednisolone (n=55) was compared to low dose (n=44) and no steroid use (n=34). Patients were also either given Chinese herb or no Chinese herb.

B. Patients with SARS not treated with corticosteroid (N=204)

**References**

1.	Gao et al. [Clinical investigation of outbreak of nosocomial severe acute respiratory syndrome]. Zhongguo Wei Zhong Bing Ji Jiu Yi Xue 2003;15(6):332-5.

2.	Zhou et al. [Epidemiologic features, clinical diagnosis and therapy of first cluster of patients with severe acute respiratory syndrome in Beijing area]. Zhonghua Yi Xue Za Zhi 2003;83(12):1018-22.

3.	Wu et al. [Clinical features of 96 patients with severe acute respiratory syndrome from a hospital outbreak]. Zhonghua Nei Ke Za Zhi 2003;42(7):453-7.

4.	Meng et al. [Clinical features of severe acute respiratory syndrome in forty-one confirmed health care workers]. Zhonghua Yu Fang Yi Xue Za Zhi 2003;37(4):236-9.

5.	Li et al. [Clinical analysis of pediatric SARS cases in Beijing]. Zhonghua Er Ke Za Zhi 2003;41(8):574-7.

6.	Li GQ, Zhang YH. [Clinical features of 77 patients with severe acute respiratory syndrome]. Zhongguo Wei Zhong Bing Ji Jiu Yi Xue 2003;15(7):404-7.

7.	Gao et al. [Analysis of relation between the usage of corticosteroid in treatment and arthralgia as a sequela of SARS patients]. Zhongguo Wei Zhong Bing Ji Jiu Yi Xue 2004;16(5):277-80.

8.	Jiang et al. [Effect of integrative Chinese and western medicine on T-lymphocyte subsets in treating patients with severe acute respiratory syndrome]. Zhongguo Zhong Xi Yi Jie He Za Zhi 2004;24(6):514-6.

9.	Liu et al. [Management of critical severe acute respiratory syndrome and risk factors for death]. Zhonghua Jie He He Hu Xi Za Zhi 2003;26(6):329-33.

10.	Xiao et al. [Glucocorticoid-induced diabetes in severe acute respiratory syndrome: the impact of high dosage and duration of methylprednisolone therapy]. Zhonghua Nei Ke Za Zhi 2004;43(3):179-82.

11.	Xu et al. [Clinical therapy of severe acute respiratory syndrome: 38 cases retrospective analysis]. Zhongguo Wei Zhong Bing Ji Jiu Yi Xue 2003;15(6):343-5.

12.	Li et al. [Retrospective analysis of the corticosteroids treatment on severe acute respiratory syndrome (SARS)]. Beijing Da Xue Xue Bao 2003;35 Suppl:16-8.

13.	Li et al. [Factors of avascular necrosis of femoral head and osteoporosis in SARS patients' convalescence]. Zhonghua Yi Xue Za Zhi 2004;84(16):1348-53.

14.	Wang et al. [The COX regression analysis on the use of corticosteroids in the treatment of SARS]. Zhonghua Yi Xue Za Zhi 2004;84(13):1073-8.

15.	Huo et al. [The clinical characteristics and outcome of 45 early stage patients with SARS]. Beijing Da Xue Xue Bao 2003;35 Suppl:19-22.

16.	Liu et al. [Clinical features and therapy of 106 cases of severe acute respiratory syndrome]. Zhonghua Nei Ke Za Zhi 2003;42(6):373-7.

17.	Cao et al. [Clinical diagnosis, treatment and prognosis of elderly SARS patients]. Zhongguo Yi Xue Ke Xue Yuan Xue Bao 2003;25(5):547-9.
