# Supplementary material for: SARS: Systematic Review of Treatment Effects
Source: PLoS Med. 2006 Sep 12;3(9):e343. doi: 10.1371/journal.pmed.0030343 (PMC1564166; doi:10.1371/journal.pmed.0030343)
Supplement: Supplementary file 9 [file pmed.0030343.st009.doc]

**Table S9**. Results of Treatment within SARS Patients (Chinese Literature)

| **Treatment of interest** | **Dose** | **Authors’ observations/inference** | **Critical appraisal** | **Conclusion** | **Reference ID** |
| --- | --- | --- | --- | --- | --- |
| **Ribavirin** | 8 mg/kg Q8Hr for 18.6 +/- 5.4 days | Death: 1/77. Combined ribavirin and corticosteroid is effective treatment. Corticosteroid use may prevent SARS patients from developing pulmonary fibrosis. | All 77 patients are from the same period and same hospital. Cannot determine effectiveness of separate treatments due to combined treatment and lack of a control group | Inconclusive | (1) |
|  | Not specified | Death 0/15. SARS is mild in children. | All children given ribavirin, steroids and IgG. The effect of treatment on outcome cannot be evaluated without a control group. | Inconclusive | (2) |
|  | Not specified | Death: 2/41. Elevated ALT in 27/41 and occurred in the 3^rd^ and 4^th^ week. May be related to treatment. Middle or low dosage of steroid was reasonable to be used as early as possible. | Ribavirin and corticosteroids were given together. The effect of treatment on outcome cannot be evaluated without a control group. | Inconclusive | (3) |
|  | 800 mg/d for 5 days | Death: 1/96. Corticosteroids, human gamma globulin, interferon-a, and antiviral drugs may be some benefit in shortening clinical course | Clinical illness in patients (mostly hospital staff) is described but the effect of treatment cannot be evaluated. | Inconclusive | (4) |
|  | Dose not specified | Death: 2/29 due to ARDS in patients who were over age 50 and received no corticosteroid or antiviral treatment. Combinational treatment of antiviral, preventative antibacterial and corticosteroid therapy may reduce the mortality of SARS. | First cohort of patients in Beijing is described. Results are observational and not evaluated with a control group. | Inconclusive | (5) |
|  | 1g then 0.5g ever 6 hr for 4 d. Then 0.5 g every 8 h for 10-14 d | Death: 4/43. Slowed heartbeat in 6/41 which was attributed to use of ribavirin. Combination of treatments was efficacious to a certain extent. | Cannot determine success or harm of any treatment or the treatment regimen due to lack of a control group. | Inconclusive | (6) |
| **Corticosteroids** | 80-640 mg/day for 12-35 days. | 26/30 experienced arthralgia symptoms during convalescence. Dose effect seen between severity of arthralgia and total dosage of corticosteroids. Timing effect between severity of arthralgia and length of duration of use of corticosteroids. | Arthralgia was a patient’s description of pain in joints lasting one day, physical exam, MRI and QUS. Need more information on how the 30 cases were chosen. Need a control group not treated with steroids to determine if arthralgia is a symptom of SARS or effect of treatment. | Inconclusive | (7) |
|  | High dose: >1400 mg or low dose: <1400mg | After low dose or high dose treatment with corticosteroids the CD3, CD4 and CD8 counts increased. The counts of those not treated with steroids did not increase. No dose effect seen because no difference in T-lymphocyte count between high dose and low dose groups. | T-lymphocyte levels were measured at baseline and after treatment and compared by IFA. Patients were retrospectively divided into 3 groups based on steroid dose. Effect of steroids cannot be evaluated with certainty because some of the group that did not receive steroids were treated with Chinese herbs and herb treatment was associated with an increase in T-lymphocytes. | Inconclusive | (8) |
|  | 80-500 mg/d for 3-4 wks then reduced to half this dose every 3-4 days | Death: 6/37 Ventilation relieved dyspnea and SpO2 in severe SARS patients. It may also help to reduce the dosage and duration of corticosteroid use. Thrombocytopenia was the main factor for patient death. Older age and underlying diseases were other factors. | Cannot determine if treatment was effective due to lack of control group not treated with steroids. | Inconclusive | (9) |
|  | Not specified | Of the 91 cases with no history of diabetes, 33 (36%) developed diabetes after corticosteroid treatment. Both over-dose and long duration of using corticosteroid leads frequently to diabetes. The maximal daily amount of use is highly related to corticosteroid induced diabetes. | Definition of diabetes was fasting plasma glucose (FPG) => 7mmol/L twice or more after administration of glucocorticocoid. Dosage of methylprednisolone was significant in a model that adjusted for age and sex, as well as % comparison. P values reported, although RR are reported without CI’s. | Possible harm | (10) |
|  | 80-50 mg/d for 3-4 wk | Death: 8/38 severe cases, 4 had underlying diseases. All who died appeared not to respond to corticosteroid. Appropriate use of corticosteroid plus ventilation is recommended to severe SARS patients. | Corticosteroid use did not appear to make a difference in outcome. Patients were also treated with IgG and an antiviral called Austavir with no response. | Inconclusive | (11) |
|  | 48-500 mg/d-1 | 4/43 patients recovered with no steroids. Following primary doses, pulse corticosteroid doses were effective in 22/25 steroid- treated patients. 14 patients were non-responsive to primary dose. | All patients also received thymosin. Cannot determine that improvement after pulse dosage of steroid is entirely due to treatment. | Inconclusive | (12) |
|  | 80-640 mg/d | 12/40 cases had necrosis of the femoral head (left3, right 0, both 9) Incidence of avascular necrosis of femoral head and osteoporosis is higher in convalescent SARS patients then general pop., which may be caused by corticosteroid treatment in earlier stage of disease. Significant factors for necrosis are presentation at late phase, steroid dose, pulsed dose. Osteoporosis related to age, pulse treatment, total dosage, duration of corticosteroid use and exercise activity post treatment. | AVN was measured by MRI. Osteoporosis was measured by QUS. Only 36/40 had IgG confirmation of SARS. Statistical analysis showed serum IgG was not related to AVN and osteoporosis. Control group of those without confirmed SARS may not be large enough to detect AVN caused by disease rather than treatment. | Possible harm | (13) |
|  | Not specified | Initiation of GCS from day 5-7 had lowest RR (0.282, 95% CI of 0.043-1.828) Corticosteroid is effective for SARS treatment but the appropriate dosage and timing seem critical to reduce the risk of death. Use of corticosteroids in patients who have comorbidities should be cautious. | All reported probable cases in Beijing were reviewed. No apparent difference between steroid group and no steroid group in terms of age and time from onset to hospitalization. RR’s and CI’s were computed for dosage, time of initiation and presence of co-morbidities. Results not significant for difference. | Inconclusive | (14) |
|  | 1.5-6mg/kg-1/d-1 or 10-15 mg/kg-1/d-1 | Fever declined after Methylprednisolone but 12/45 patents had relapse in fever. MP use showed improvement on x-ray diffusion. | 10 patients had elevated blood sugar levels after treatment with corticosteroids although the paper did not discuss this effect. Cannot determine effect of steroids due to lack of control group | Inconclusive | (15) |
|  | 40-160 mg/d | Death: 6/106. Oxygen support and small dosage of corticosteroid treatment is effective. Detecting a decrease of CD4 counts may facilitate early diagnosis of SARS. | Cannot determine if treatment was effective due to lack of control group not treated with steroids. | Inconclusive | (16) |
|  | 80-160 mg/d for 3, 5 days then 40 mg/d for 2-3 days | Death: 1/96. Corticosteroids, human gamma globulin, interferon-a, and antiviral drugs may be some benefit in shortening clinical course. Temperature decreased from 38.4 +/-1C to 36.9 +/- 0.7C after corticosteroid treatment. | Clinical illness in patients (mostly hospital staff) is described but the effect of treatment cannot be evaluated. | Inconclusive | (4) |
|  | 80-240 mg/d | Death: 2/41. Elevated ALT in 27/41 and occurred in the 3^rd^ and 4^th^ week. May be related to treatment. Middle or low dosage of steroid was reasonable to be used as early as possible. | Ribavirin and corticosteroids were given together and therefore cannot determine effect of either one on outcome. | Inconclusive | (3) |
|  | Not specified | Death 0/15. SARS is mild in children. | All children given ribavirin, steroids and IgG. No information on treatment effect | Inconclusive | (2) |
|  | Not specified | Death 7/24 elderly patients. 15/24 had underlying disesase. Age >60 had more severe SARS course than those < 60 years. | This study was to compare illness in older patients with that in younger patients. Effect of corticosteroid treatment cannot be evaluated. | Inconclusive | (17) |
| **Interferon-alpha** | Not specified | Death: 1/96. Corticosteroids, human gamma globulin, interferon-a, and antiviral drugs may be some benefit in shortening clinical course | Clinical illness in patients (mostly hospital staff) is described but the effect of treatment cannot be evaluated. | Inconclusive | (4) |
| **Immunoglobulin** | Not specified | Death: 1/96. Corticosteroids, human gamma globulin, interferon-a, and antiviral drugs may be some benefit in shortening clinical course | Clinical illness in patients (mostly hospital staff) is described but the effect of treatment cannot be evaluated. | Inconclusive | (4) |
|  | Not specified | Death 0/15. SARS is mild in children. | All children given ribavirin, steroids and IgG. No information on treatment effect | Inconclusive | (2) |

**References**

1.	Li GQ, Zhang YH. [Clinical features of 77 patients with severe acute respiratory syndrome]. Zhongguo Wei Zhong Bing Ji Jiu Yi Xue 2003;15(7):404-7.

2.	Li Z et al. [Clinical analysis of pediatric SARS cases in Beijing]. Zhonghua Er Ke Za Zhi 2003;41(8):574-7.

3.	Meng et al. [Clinical features of severe acute respiratory syndrome in forty-one confirmed health care workers]. Zhonghua Yu Fang Yi Xue Za Zhi 2003;37(4):236-9.

4.	Wu et al. [Clinical features of 96 patients with severe acute respiratory syndrome from a hospital outbreak]. Zhonghua Nei Ke Za Zhi 2003;42(7):453-7.

5.	Zhou et al. [Epidemiologic features, clinical diagnosis and therapy of first cluster of patients with severe acute respiratory syndrome in Beijing area]. Zhonghua Yi Xue Za Zhi 2003;83(12):1018-22.

6.	Gao et al. [Clinical investigation of outbreak of nosocomial severe acute respiratory syndrome]. Zhongguo Wei Zhong Bing Ji Jiu Yi Xue 2003;15(6):332-5.

7.	Gao et al. [Analysis of relation between the usage of corticosteroid in treatment and arthralgia as a sequela of SARS patients]. Zhongguo Wei Zhong Bing Ji Jiu Yi Xue 2004;16(5):277-80.

8.	Jiang et al. [Effect of integrative Chinese and western medicine on T-lymphocyte subsets in treating patients with severe acute respiratory syndrome]. Zhongguo Zhong Xi Yi Jie He Za Zhi 2004;24(6):514-6.

9.	Liu et al. [Management of critical severe acute respiratory syndrome and risk factors for death]. Zhonghua Jie He He Hu Xi Za Zhi 2003;26(6):329-33.

10.	Xiao et al. [Glucocorticoid-induced diabetes in severe acute respiratory syndrome: the impact of high dosage and duration of methylprednisolone therapy]. Zhonghua Nei Ke Za Zhi 2004;43(3):179-82.

11.	Xu et al. [Clinical therapy of severe acute respiratory syndrome: 38 cases retrospective analysis]. Zhongguo Wei Zhong Bing Ji Jiu Yi Xue 2003;15(6):343-5.

12.	Li et al. [Retrospective analysis of the corticosteroids treatment on severe acute respiratory syndrome (SARS)]. Beijing Da Xue Xue Bao 2003;35 Suppl:16-8.

13.	Li et al. [Factors of avascular necrosis of femoral head and osteoporosis in SARS patients' convalescence]. Zhonghua Yi Xue Za Zhi 2004;84(16):1348-53.

14.	Wang et al. [The COX regression analysis on the use of corticosteroids in the treatment of SARS]. Zhonghua Yi Xue Za Zhi 2004;84(13):1073-8.

15.	Huo et al. [The clinical characteristics and outcome of 45 early stage patients with SARS]. Beijing Da Xue Xue Bao 2003;35 Suppl:19-22.

16.	Liu et al. [Clinical features and therapy of 106 cases of severe acute respiratory syndrome]. Zhonghua Nei Ke Za Zhi 2003;42(6):373-7.

17.	Cao et al. [Clinical diagnosis, treatment and prognosis of elderly SARS patients]. Zhongguo Yi Xue Ke Xue Yuan Xue Bao 2003;25(5):547-9.
