# Supplementary material for: SARS: Systematic Review of Treatment Effects
Source: PLoS Med. 2006 Sep 12;3(9):e343. doi: 10.1371/journal.pmed.0030343 (PMC1564166; doi:10.1371/journal.pmed.0030343)
Supplement: Supplementary file 10 [file pmed.0030343.st010.doc]

**Table S10**. Description of Studies of ARDS or ALI

| **Treatment of interest** | **Country** | **Study design** | **Comparison?** | **Study N** | **Steroids?** | **Outcomes Measured** | **Reference ID** |
| --- | --- | --- | --- | --- | --- | --- | --- |
| Corticosteroids | USA | RCT | Y^A^ | 99 | Yes | Death, infectious complications | (1) |
|  | USA | RCT | Y^B^ | 24 | Yes | Death, infectious complications, ventilation | (2) |
|  | USA | RCT | Y^C^ | 99 | Yes | Death, infectious complications, ARDS | (3) |

A. methylprednisolone (N=50) vs. placebo (N=49)

B. methylprednisolone (N=16) vs. placebo (N=8)

C. methylprednisolone (N=39) vs. placebo (N=42)

**References**

1. Bernard et al. High-dose corticosteroids in patients with the adult respiratory distress syndrome. N Engl J Med 1987;317(25):1565-70.

2. Meduri et al. Effect of prolonged methylprednisolone therapy in unresolving acute respiratory distress syndrome: A randomized controlled trial. Journal of the American Medical Association 1998;280(2):159-65.

3. Weigelt et al. Early steroid therapy for respiratory failure. Arch Surg 1985;120(5):536-40.
