# Supplementary material for: SARS: Systematic Review of Treatment Effects
Source: PLoS Med. 2006 Sep 12;3(9):e343. doi: 10.1371/journal.pmed.0030343 (PMC1564166; doi:10.1371/journal.pmed.0030343)
Supplement: Supplementary file 11 [file pmed.0030343.st011.doc]

**Table S11. Results of Treatment of ARDS or ALI**

| **Treatment of interest** | **Steroid Dose** | **Author’s observations** | **Critical Appraisal** | **Conclusion** | **Reference ID** |
| --- | --- | --- | --- | --- | --- |
| **Corticosteroids** | MP, 30mg/kg every 6 hours for 4 doses. | Death: 30/50 vs. 31/49. Infectious complications: 8/50 v 5/49. High-dose methylprednisolone given in early course of established ARDS had no effect on survival or reversal of respiratory failure. No significant difference seen. Blood glucose levels increased in MP group (p<.01). | Good quality study of short-term MP treatment. Patients in placebo and treatment group were similar. Relatively small sample size but no significant effect of treatment demonstrated. | No benefit | (1) |
|  | MP, 2mg/kg/day for 14 days, tapered dose until day 32. | Death: 2/16 vs. 5/8. Infectious complications: 8/100 days vs. 7/100 days. Prolonged methylprednisolone significantly associated with improvement. | Small RCT, randomization may not have produced entirely equal patient groups, but some evidence for improvement of established ARDS. | Possible benefit | (2) |
|  | MP, 30mg/kg every 6 hours for 48 hours. | Death: 18/39 vs. 13/42. Steroid administration significantly increased the degree of pulmonary deterioration. Steroids do not have any beneficial effects for critically ill patients with pulmonary failure. Infectious complications: 30/39 v 18/42 | Study population was intubated and mechanically ventilated patients at risk for ARDS. Difference in mortality was not significant (P=0.177), however, credible evidence of deterioration in patients with ALI despite steroids. | No benefit | (3) |

**References**

1. Bernard et al. High-dose corticosteroids in patients with the adult respiratory distress syndrome. N Engl J Med 1987;317(25):1565-70.

2. Meduri et al. Effect of prolonged methylprednisolone therapy in unresolving acute respiratory distress syndrome: A randomized controlled trial. Journal of the American Medical Association 1998;280(2):159-65.

3. Weigelt et al. Early steroid therapy for respiratory failure. Arch Surg 1985;120(5):536-40.
